# Exploiting machine learning models to identify novel Alzheimer’s disease biomarkers and potential targets

**DOI:** 10.1038/s41598-023-30904-5

**Published:** 2023-03-27

**Authors:** Hind Alamro, Maha A. Thafar, Somayah Albaradei, Takashi Gojobori, Magbubah Essack, Xin Gao

**Affiliations:** 1 Computer Science Program, Computer, Electrical and Mathematical Sciences and Engineering Division (CEMSE), King Abdullah University of Science and Technology (KAUST), Thuwal, Saudi Arabia; 2 Computational Bioscience Research Center (CBRC), King Abdullah University of Science and Technology (KAUST), Thuwal, Saudi Arabia; 3 College of Computer and Information Systems, Umm Al-Qura University, Makkah, Saudi Arabia; 4 College of Computers and Information Technology, Taif University, Taif, Saudi Arabia; 5 Faculty of Computing and Information Technology, King Abdulaziz University, Jeddah, Saudi Arabia

**Keywords:** Computational biology and bioinformatics, Computational models, Gene regulatory networks

## Abstract

We still do not have an effective treatment for Alzheimer's disease (AD) despite it being the most common cause of dementia and impaired cognitive function. Thus, research endeavors are directed toward identifying AD biomarkers and targets. In this regard, we designed a computational method that exploits multiple hub gene ranking methods and feature selection methods with machine learning and deep learning to identify biomarkers and targets. First, we used three AD gene expression datasets to identify 1/ hub genes based on six ranking algorithms (Degree, Maximum Neighborhood Component (MNC), Maximal Clique Centrality (MCC), Betweenness Centrality (BC), Closeness Centrality, and Stress Centrality), 2/ gene subsets based on two feature selection methods (LASSO and Ridge). Then, we developed machine learning and deep learning models to determine the gene subset that best distinguishes AD samples from the healthy controls. This work shows that feature selection methods achieve better prediction performances than the hub gene sets. Beyond this, the five genes identified by both feature selection methods (LASSO and Ridge algorithms) achieved an AUC = 0.979. We further show that 70% of the upregulated hub genes (among the 28 overlapping hub genes) are AD targets based on a literature review and six miRNA (hsa-mir-16-5p, hsa-mir-34a-5p, hsa-mir-1-3p, hsa-mir-26a-5p, hsa-mir-93-5p, hsa-mir-155-5p) and one transcription factor, JUN, are associated with the upregulated hub genes. Furthermore, since 2020, four of the six microRNA were also shown to be potential AD targets. To our knowledge, this is the first work showing that such a small number of genes can distinguish AD samples from healthy controls with high accuracy and that overlapping upregulated hub genes can narrow the search space for potential novel targets.

## Introduction

Alzheimer’s disease (AD) is the most common cause of dementia, and its prevalence increases with age^1,2^. Unfortunately, no approved drugs can prevent or delay AD progression despite the array of potential targets identified for AD treatment. Also, many potential therapies in clinical trials have failed, including the most promising Aβ-directed therapies^3,4^. The problem may be that although our knowledge of AD progression has grown, and we know the development of amyloid-β (Aβ) plaques and tau neurofibrillary tangles in the brain are hallmarks of AD, the real cause of AD is still unclear^5,6^. Nonetheless, early detection of AD is key to its treatment. Thus, researcher efforts are also directed at detecting AD using artificial intelligence (AI), machine learning (ML), and deep learning (DL) algorithms and incorporating different types of data including but not limited to: neuroimaging data^7^, non-coding RNAs^8,9^, transcriptomic data^10^, miRNAs biomarker^11^, or other genome data^12^. Another direction that researchers paid more attention to is to repurpose approved drugs to treat AD^13,14^.

Despite the caveats in our understanding of AD pathogenesis, current knowledge has allowed us to identify potential biomarkers and several proteins that may serve as effective targets in counteracting AD. Many of these biomarkers and targets are now also first determined through in silico means to minimize the considerable investments in developing novel drugs. For example, Madar et al.^15^ used 26 differentially expressed genes (DEGs), shortlisted based on p-value and analyses using the online annotation tool DAVID, to build six different classifiers that differentiate healthy and diseased samples. Then, they performed additional co-expression analyses to identify 13 of the genes as potential AD biomarkers. Perera et al.^16^, on the other hand, determined the AD DEGs and then shortlisted them using PCA, Random Forest, and Extra Tree Classifier as feature selection methods. They used the shortlisted features to build six different classifiers that differentiate healthy and diseased samples, and the feature importance scores from Random Forest and Extra Tree Classifier and correlation matrix were used to identify 14 new candidate biomarker genes for AD. Zhao et al.^17^ started by identifying the key modules associated with AD, then performed functional enrichment analysis to reveal the hub genes, which they validated by machine learning algorithms. Yu et al.^18^ determined the AD DEGs, from which hub genes were shortlisted and genes based on the LASSO feature selection method. They identified a 16 hub gene set and a 35 biomarker set based on LASSO and proposed the overlapping genes as potential AD targets through this process. These studies obtained significantly different results based on the methodologies used, but they each contribute to revealing more of the complex AD progression.

Several studies have analyzed the gene expression in different brain regions and compared the gene expression in specific brain regions to unravel the underlying biology. Here, instead, we used machine learning to pinpoint genes that feature in AD’s progression despite the differences in sample type, such as laser-captured neurons versus bulk tissue or differences in brain regions. First, we used AD DEGs to determine hub genes through six ranking algorithms (and the overlapping genes) and two feature selection methods (and the overlapping genes). We then used this to determine the gene set/s that best contribute to an AD versus healthy controls prediction task using random forests (RF) and support vector machine (SVM) classifiers for the ML models, and convolution neural networks (CNN) and deep neural networks (DNN) for DL models. The feature selection methods achieved better prediction performances than the hub gene sets, and the five overlapping genes from the feature selection methods (LASSO and Ridge algorithms) achieved an AUC = 0.979. We also explored which set of overlapping genes is more aligned with the underlying biology of AD based using DisGeNET in Enrichr and found that 7 of the top-10 enriched diseases were neurological diseases/disorders for the upregulated genes from the overlapping hub gene set. Furthermore, we conducted a literature review and found a substantial portion of the pinpointed upregulated hub genes (70%) are known AD targets.

## Materials and method

### Gene expression data

We searched for gene expression datasets of brain tissue in the Gene Expression Omnibus (GEO) database^19^ using the query: "Alzheimer* AND Homo sapiens" filtered by "Expression profiling by array" on the 2nd March 2022. We retrieved 188 entries which we sifted through. We found three datasets (GSE5281^20^, GSE48350^21^, and GSE1297^22^) generated using the same platform, in this case, Affymetrix Human Genome U133 Plus 2.0 Array, that provides gene expression data of the brain tissue of AD patients and healthy controls within the same age range. There are 253 samples (80 ADs and 173 controls) in GSE48350, 161 samples (87 ADs and 74 controls) in GSE5281, and 31 samples in GSE1297 (22 ADs and 9 controls). In total, we had 455 samples (189 ADs and 256 controls) which we used to develop our ML and DL models. We also used the GSE109887^23^ and GSE138260^24^ datasets for independent testing. GSE109887 is a brain tissue dataset from the medial temporal gyrus, containing 46 AD samples and 32 controls, and GSE138260 is a brain tissue dataset containing 17 AD samples and 19 controls. Table 1 describes the selected datasets.

**Table 1:** Description of the datasets selected for training and independent testing.

Dataset IDs	No. of Samples	Healthy controls/AD	Brain region	Female/ Male	Use
GSE5281	161	74/87	Hippocampus Entorhinal cortex Medial temporal gyrus Posterior cingulate Superior frontal gyrus Primary visual cortex	58/103	Training
GSE48350	253	173/80	Hippocampus Entorhinal cortex Superior frontal cortex Post-central gyrus	129/124	Training
GSE1297	31	9/22	Hippocampus, Entorhinal cortex	18/13	Training
GSE109887	78	32/46	Medial temporal gyrus	40/38	Independent testing
GSE138260	36	19/17	Brain Tissue	19/16 (one sample is NA)	Independent testing

Note, for dataset selection, we considered the datasets with more than 30 samples. However, several datasets have been excluded for different cases, some datasets were excluded by the ImaGEO quality control tool (elaborated on in the next section). Other datasets that we checked containing duplicates for the same individual, in the same brain region, with highly varying expression levels, were also excluded, as this reflects poorly on the quality of the provided data.

### Meta-analysis of the gene expression data

We used ImaGEO^25^, a web-based platform that integrates and performs meta-analyses of multiple GEO datasets. We aimed to combine the same experimental condition across different studies to increase the sample size and statistical power. Thus, we established the ImaGEO meta-analysis as an effect size method. Then, we selected the fixed-effect model parameter for the effect size estimation to identify the genes with the most potent effect in the selected datasets with an adjusted p-value < 0.05, with only 10% missing values allowed. Next, we used ImaGEO to integrate all three GEO datasets (GSE5281, GSE48350, and GSE1297), perform background correction, normalization, batch effect correction, and apply initial differential expression analysis. Through this process, ImaGEO generated an integrated matrix with 924 genes as the potential DEGs, which we used in subsequent analyses. To define our features for the ML models, we downloaded the matrix file provided by ImaGeo for each of the three datasets. We then used R^26^, an open-source statistical and scientific programming language for data analysis, to integrate the matrices, creating the full matrix containing gene expression for all the samples. After that, we selected the DEGs from this full matrix to build the final matrix with 445 samples and 924 features (DEGs).

### Identifying hub genes using the PPI network

Hub genes are regularly used to zoom in on the subset of DEGs that would best discern the diseased samples from the healthy control. Thus, we used STRING^27^, a biological database and web resource of known and predicted PPI (http://string-db.org), to explore the interactions between the DEGs. Next, we used the Cytoscape software^28^ (version 3.9.1) (https://cytoscape.org/) to visualize the network, and we utilized the cytoHubba plugin in Cytoscape to identify the hub genes in the PPI network using several ranking methods.

CytoHubba^29^ provides different algorithms for node ranking, including local and global methods. The local rank method considers the relationship between the node and its direct neighbors, while the global method examines the relationship between the node and the entire network. We used six ranking algorithms to determine the hub genes, including three local ranking algorithms (Degree, Maximum Neighborhood Component (MNC), Maximal Clique Centrality (MCC)), and three global ranking algorithms (Betweenness Centrality (BC), Closeness Centrality, and Stress Centrality). The degree of a node *v* is the number of its adjacent nodes. MNC is the size of the maximum connected component of N(*v*), where the neighborhood N(*v*) is the set of nodes adjacent to *v* and does not contain node v. Stress centrality measures the absolute number of shortest paths, while betweenness centrality measures the fraction of the shortest paths passing through a node. Finally, closeness centrality indicates how close a node is to all other nodes in the network, calculated as the average of the shortest path length from the node to every other node in the network. Finally, we used the top-100 genes from each ranking method to develop ML/DL models.

### Identifying the subset of DEGs that best discern the diseased samples from the healthy control using feature selection models

To identify the subset of DEGs that best differentiate the diseased samples from the healthy controls, we applied feature selections algorithms, including LASSO regression (Least Absolute Selection and Shrinkage Operator)^30^ and Ridge regression^31^ that select the best features in high-dimensional data (i.e., provide a principled way to reduce the number of features in a model). These algorithms assign an importance score to each feature based on the feature's ability to predict the correct label. Briefly, LASSO regression eliminates many features and reduces overfitting in the linear model, while Ridge regression minimizes the impact of features that are not important in predicting the sample’s label. LASSO involves a penalty factor that determines how many features are retained; using cross-validation (CV) to choose the factor helps assure that the model will generalize well to future data samples. We implemented LASSO and Ridge algorithms using Python programming language and the Sikitlearn library. To apply the LASSO regression feature selection, we first need to tune the alpha (α) hyperparameter to make a suitable regression model and obtain the best performance. Thus, we utilized grid search for an α parameter by applying GridSearchCV using fivefold CV and repeated the process five times. Next, we fed the sample matrix with all the features (924 DEGs) into LASSO logistic regression. The model then allows us to select the best subset of features based on a threshold important score. We evaluated the ability of each subset chosen to differentiate between AD and healthy samples by calculating the AUC scores using our ML/DL models and then selected the subset with the highest AUC. We applied similar steps to the Ridge algorithm. At the end of this process, the α values selected were 0.01 and 0.99 for LASSO and Ridge, respectively.

### Developing ML and DL models

We developed several ML and DL models to distinguish between the AD samples and the healthy controls. We implemented RF and SVM classifiers for the ML models and CNN and DNN for the DL models.

For the ML models, we created a search space for each model for parameter optimization to find the best combination of parameters. Therefore, we used a randomized search followed by a grid search algorithm for hyperparameter optimization in RF and SVM. We created a grid of hyperparameters for the randomized search and then trained/tested our models using random hyperparameter combinations. Next, the best parameters identified through the randomized search are subjected to a grid search to find the optimal parameter combinations.

For the DL models, we only applied the grid search in our hyperparameter search space. We implemented DNN, a neural network with four hidden layers with unit sizes (64, 64, 32, 32), followed by a dense layer. We set the batch size to 128 and trained 500 epochs. In the same way, we implemented CNN, a convolutional neural network using two 1D-CNN layers with filter sizes 64, followed by two dense layers with unit sizes 32. We used max-pooling and flatten layers between the CNN and the dense layer. We trained the model with batch size = 128 and 500 epochs. Table 2 summarizes the tested values and bold font indicates the selected values.

**Table 2:** Parameter search space for optimizing RF, SVM, DNN and CNN models.

Model	Hyperparameters	Search space
RF	Max_depth	[10,20,30,**40**,50,60,70,80,90,100]
	N_estimators	[100,**200**,300,400,500,600]
	Min_samples_leaf	[**1**, 4, 6, 8, 12]
SVM	Gamma	[‘scale’, **‘auto**’]
	Kernel	[‘linear’, ‘poly’, ‘**rbf’**]
	C	[0.05, 0.25, 0.5**, 1.0**, 1.5, 2.0]
DNN	Activation function	['softmax', 'relu', '**tanh**', 'sigmoid', 'linear']
	Optimizers	['SGD', 'RMSprop','Adam', '**Nadam**']
	Node size in each layer	[**32, 64**, 128, 256]
	Batch size	[8, 16, 32, 64, **128**, 256]
	Epochs	[50, 100, 200, **500**, 1000]
CNN	Activation function	['softmax', 'relu', '**tanh**', 'sigmoid', 'linear']
	Optimizers	['SGD', 'RMSprop','Adam', '**Nadam**']
	Filters	[**32, 64**, 128, 256]
	Batch size	[8, 16, 32, 64, **128**, 256]
	Epochs	[50, 100, 200, **500**, 1000]

The optimal parameter values are in [bold].

To evaluate these models, we used five-fold-cross-validation (CV), where the data is divided into five subsets using the StratifiedKFold method, which ensures that all subsets include the same percentage of positive and negative samples (i.e., AD and controls). The process is implemented (repeated) five times by keeping one subset for testing and using all the remaining sets for training. The AUC score of each fold is calculated, which we used to report the average AUCs.

### Bioinformatics analyses

For the bioinformatics analyses, we used DisGeNET housed in the comprehensive gene set analyses tool, Enrichr^32^. We ranked the results based on the Odds Ratio. We also used miRNet^33^ with a network degree filter cutoff of 5.0 for all network nodes to determine the key set of microRNA and transcription factors associated with the upregulated genes among the 28 overlapping hub genes.

## Results and discussion

### The study design

**Figure 1:**
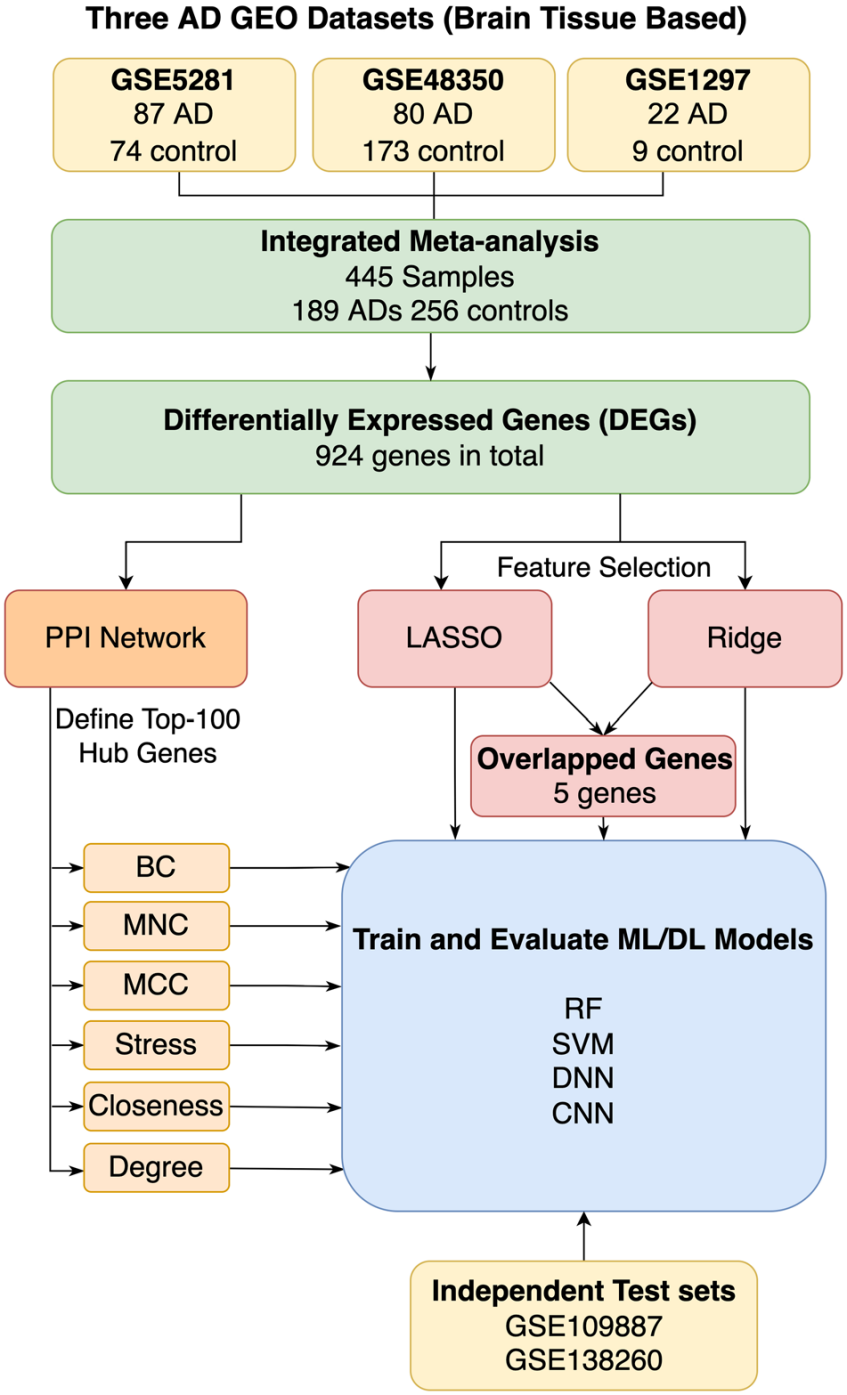
The study workflow consists of two key paths, via ranking algorithms and feature selection methods.

The workflow of our study incorporates six main steps, as depicted in Fig. 1. First, we downloaded three brain tissue-based datasets from the National Center of Biotechnology Information-GEO (NCBI-GEO) Datasets^34^ accessed by March 2022. The details and statistics of these datasets are given in Table 1. Thus we obtained a total of 445 samples consisting of 189 ADs and 256 controls. Second, after combining all data samples, we used them in an integrated meta-analysis. Therefore, the analysis identified 2915 DEGs in GSE5281, 163 DEGs in GSE48350, and 4 DEGs in GSE1297. Third, we utilized the ImaGEO tool to integrate and identify the DEGs of the three integrated datasets (i.e., the overlapped DEGs that are found in the three datasets). Fourth, we identified the hub genes using six graph ranking algorithms. Fifth, we identified the most significant DEGs using other feature selection methods. The sixth step is to build and evaluate ML/DL models using different sets of features (i.e., DEGs) generated from the latter two steps. Each step is explained later in more detail in the corresponding subsection. Finally, we tested our best models using independent datasets.

### Identifying DEGs between brain samples from AD patients and healthy aged controls

**Figure 2:**
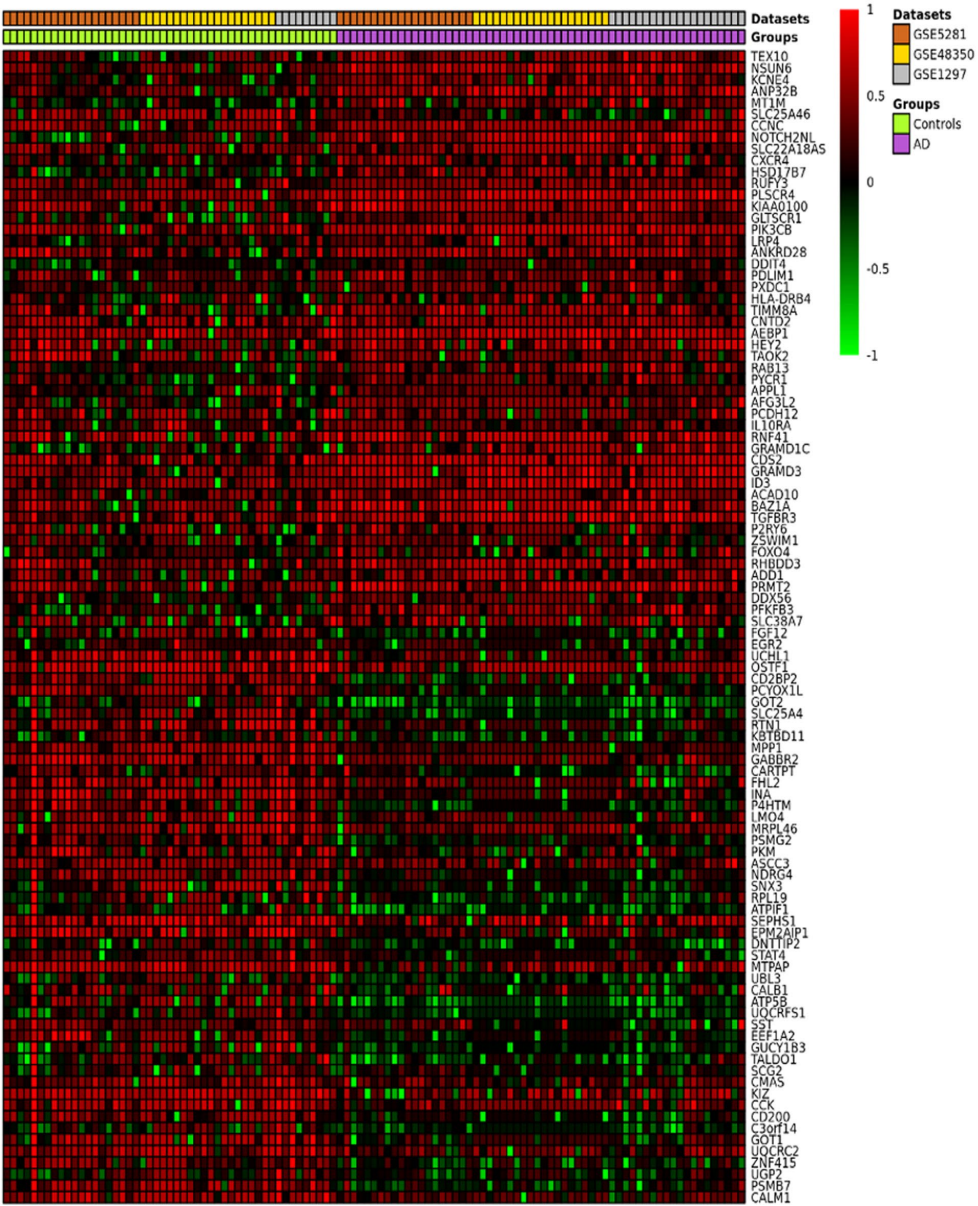
An ImaGEO generated heatmap of the top-100 DEGs (Red represents the relative upregulated gene expression; green represents the relative downregulated gene expression; black represents no significant change in gene expression).

The ImaGEO tool's quality control test shows the data used in this study is of good quality. For the integrated datasets, the ImaGEO tool’s meta-analysis further identified 924 DEGs, including 512 upregulated DEGs and 403 down-regulated DEGs. Supplementary Table S1 provides the complete list of DEGs. A visual representation of the top-100 DEGs in a heatmap shows that the expression of more of the genes in the AD group is consistently upregulated in all the samples compared to the healthy aged controls (see Fig. 2, purple represents the AD samples, and the green represents the control samples from the healthy aged individuals, annotated at the top of each plot). Also, about 50% of these clearly down-regulated genes in the AD group are consistently upregulated in the control samples. On the other hand, most of the genes upregulated in the so-called ‘healthy aged controls’ were not consistently upregulated in all the samples, and expression levels varied dramatically across samples. This observation is not too surprising as Berchtold et al.^21^, amongst others, reported age-dependent changes in gene expression in the brain. Specifically, the aged brain in both sexes increases immune activity, but it is proportionally in the female brain, and the male brain suffers a global decrease in catabolic and anabolic capacity. These sex and age-dependent changes in gene expression in the brain are thought to set the balance between neurodegeneration and compensatory mechanisms in the brain.

### Determining the subset of DEGs that can serve as features to build the ML/DL models

We determined the features needed to build the ML/DL models using: 1/ various ranking algorithms to identify the hub genes and 2/ feature selection methods to determine the gene sets that best contribute to the prediction task.

Briefly, we used the 924 DEGs to construct a PPI network using STRING. To remove all nodes with less than five connected edges, we filtered the PPI network using a threshold of five degrees, which generated a network consisting of 863 nodes (i.e., genes) and 4720 edges (i.e., direct physical PPI). This network was then fed into Cytoscape software^28^ to visualize and determine the hub genes using the cytoHubba plugin. As a result, we obtained the top-100 hub genes for six topological ranking algorithms, including Degree, Betweenness Centrality (BC), Maximum Neighborhood Component (MNC), Maximal Clique Centrality (MCC), Closeness Centrality, and Stress Centrality (see Supplementary Table S2). Surprisingly, we found that all six ranking algorithms commonly identified only 28 of the hub genes (ATP5B, ATP5C1, CYCS, NDUFA4', ACO2, NHP2, RPS7, FAU, EIF2S1, CCT7, UBC, PTEN, PSMD4, PSMD7, GOT2, SNAP25, MAPT, BDNF, NTRK2, SNCA, APP, JUN, IGF1, MAP2K1, RAF1, CDC42, ENO2, JAK2), and if we exclude the MCC list, the genes commonly identified by the ranking algorithms increase to 53 hub genes. Finally, we used these hub gene lists to develop computational models that could differentiate between AD samples and the healthy control samples.

We also applied the LASSO and Ridge logistic regression feature selection methods using the 924 DEGs. We tested several threshold values to select the best subset of features for both methods. Utilizing LASSO logistic regression, we obtained gene sets of 71, 27, and 8 by specifying the important scores higher than 0.01, 0.02, and 0.03, respectively. Similarly, for the Ridge algorithm, we obtained sets of 80, 41, and 26 genes by specifying the important scores higher than 0.05, 0.06, and 0.07, respectively.

### Evaluating the prediction performance of the ML and DL models

We evaluated the changes in the prediction performances of the two ML (RF and SVM) and the two DL (DNN and CNN) models when fed different sets of the features determined by the six ranking algorithms (hub genes) and the two feature selection methods separately (see Fig. 1).

#### Prediction performances using hub genes determined by diverse ranking algorithms

We constructed RF, SVM, DNN, and CNN models using genes identified through the six ranking algorithms separately. We iteratively added ten of the top-100 ranked genes for each ranking algorithm to evaluate the models. Briefly, the evaluation process is as follows: first, we obtained the featured hub genes list based on the MNC algorithm, sorted using the MNC score. We evaluated the performance of the top genes by iteratively adding ten by ten genes each time. That is, for training and testing, we added the 10 top-ranked genes, used the cross-validation method, and reported the averaged AUC score as described in section "Identifying hub genes using the PPI network". Then, we added the next set of 10 genes, making it the top-20 genes, and applied the same evaluation process to obtain the averaged AUC. We repeated this process until the complete list of the top-100 genes was fed into the RF classifier. Note, we performed the same procedure for all ranking lists separately.

Table 3 provides the results for each experiment of the evaluation process. For each model, we underline the best prediction performance for each ranking method, then the bold and italic indicate the best and second-best of these results. The results show that the BC ranking algorithm consistently appeared among the best and second-best of these results for all the models, followed by Stress Centrality and Degree. On the other hand, the MCC list generally produced the worst performances for all the models, except DNN.

**Table 3:** Based on six topological ranking algorithms, the prediction performances for RF, SVM, CNN, and DNN in terms of AUC for top-ranked DEGs (increased by 10).

RF										
Ranking	top10	top20	top30	top40	top50	top60	top70	top80	top90	top100
BC	0.773	0.796	0.8286	0.8454	0.8599	0.8604	*0.8696*	**0.8735**	0.8659	0.8642
MNC	0.7576	0.8176	0.8272	0.847	0.849	0.8523	0.8607	0.8592	0.8666	0.8651
MCC	0.746	0.8277	0.8513	0.8469	0.8493	0.856	0.8507	0.8427	0.8509	0.8555
Stress	0.7603	0.8028	0.8284	0.8428	0.8613	0.855	0.8641	0.8628	0.8638	0.8658
Close	0.7684	0.7889	0.8107	0.8326	0.8403	0.8502	0.8469	0.859	0.8645	0.8661
Degree	0.7576	0.8176	0.8344	0.8436	0.8541	0.848	0.8556	0.8583	0.8671	0.8669

SVM										
Ranking	top10	top20	top30	top40	top50	top60	top70	top80	top90	top100
BC	0.7064	0.7497	0.7636	0.799	0.7978	0.8101	0.8114	*0.8246*	**0.8247**	0.8183
MNC	0.7061	0.7518	0.7621	0.7843	0.787	0.781	0.7955	0.8032	0.8119	0.8104
MCC	0.7029	0.739	0.7467	0.7476	0.7519	0.7692	0.7719	0.7863	0.7815	0.7841
Stress	0.6995	0.7406	0.7607	0.786	0.81	0.7985	0.8009	0.8123	0.8081	0.817
Close	0.6845	0.7339	0.7326	0.7859	0.781	0.7922	0.8002	0.7979	0.8049	0.8062
Degree	0.7061	0.7518	0.7615	0.782	0.7929	0.7922	0.7871	0.7921	0.8129	0.8105

CNN										
Ranking	top10	top20	top30	top40	top50	top60	top70	top80	top90	top100
BC	0.7431	0.8324	0.8511	0.8353	0.7855	0.843	0.8281	0.8489	*0.8524*	0.8465
MNC	0.7465	0.8245	0.836	0.7878	0.8397	0.8449	0.8369	0.8011	0.8336	0.8277
MCC	0.7225	0.7853	0.7954	0.7904	0.8193	0.809	0.8153	0.796	0.7922	0.8123
Stress	0.7337	0.7652	0.8381	0.8012	0.8352	0.8443	0.8453	**0.853**	0.8502	**0.853**
Close	0.7507	0.7529	0.7889	0.8109	0.7925	0.8131	0.8254	0.8376	0.8301	0.8422
Degree	0.7465	0.8245	0.8339	0.808	0.8494	0.824	0.8373	0.8121	0.8483	0.8234

DNN										
Ranking	top10	top20	top30	top40	top50	top60	top70	top80	top90	top100
BC	0.7908	0.8262	*0.8793*	**0.8955**	0.8721	0.8641	0.8727	0.8641	0.8622	0.8725
MNC	0.7819	0.8631	0.8413	0.853	0.8672	0.8643	0.8529	0.8425	0.8564	0.8546
MCC	0.756	0.8101	0.8629	0.8472	0.8756	0.8766	0.8491	0.8498	0.8622	0.84
Stress	0.7958	0.8223	0.8546	0.863	0.8736	0.8621	0.861	0.8749	0.8665	0.8764
Close	0.7937	0.812	0.8552	0.8372	0.8429	0.8505	0.8412	0.8598	0.8613	0.8556
Degree	0.7819	0.8631	0.8698	0.8653	0.8613	0.8495	0.8397	0.8449	0.8428	0.8585

The underline indicates the highest AUC achieved for each ranking algorithm. The Bold and Italic values suggest the best and second-best performing ranking algorithms.

Focussing on the models, we find that the RF and DNN models clearly outperformed other models. Specifically, the DNN achieved the best prediction performance with an AUC of 0.8955 using the top-40 genes of the BC ranking list. On the other hand, the best prediction performance for RF is an AUC of 0.8735, achieved with the top-80 genes of the BC ranking list.

#### Prediction performances of the models using genes determined by feature selection algorithms

**Figure 3:**
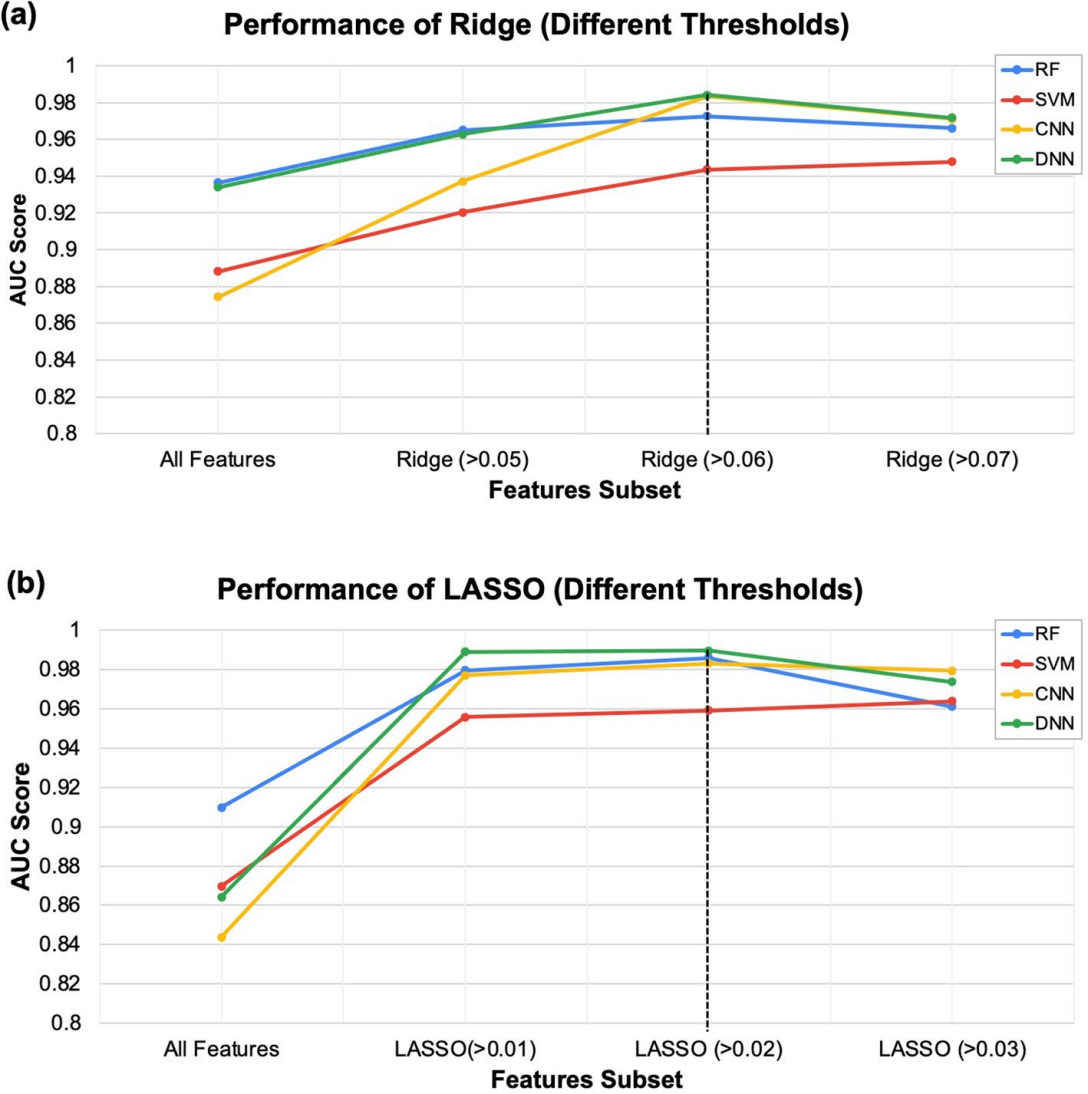
The prediction performance of the list of DEGs selected by (**a**) LASSO (L) and (**b**) Ridge (R) regression algorithms at multiple thresholds for the importance scores.

We evaluated the RF, SVM, DNN, and CNN models separately based on two feature selection algorithms, LASSO and Ridge. We evaluated the prediction performances at multiple thresholds for the importance scores. Figure 3 provides the prediction performances achieved using the features selection algorithms. First, all the AUC results are better when applying Ridge or LASSO feature selection methods than when feeding all 924 DEGs to ML and DL models, indicating that filtering the features by removing some of them with low importance scores improves the results. Second, the Ridge algorithms achieved the best results when using the important score > 0.06 (Fig. 3 a). While the best results were achieved for the LASSO algorithms when using the important score > 0.02 (Fig. 3 b). Another observation is that all ML and DL prediction performances were significantly higher when applying Ridge and LASSO features than when the hub genes were applied. The high AUC demonstrates that both the LASSO and Ridge algorithms captured the significant DEGs that allow the classifiers to distinguish the AD samples from the samples belonging to the healthy aged controls. Both the LASSO and Ridge algorithms achieved high AUCs of 0.9896 and 0.9841, respectively, with the DNN model, outperforming all the other models. The lists of genes are provided in Supplementary Table S3.

#### Prediction performances using overlapping hub genes determined by ranking and feature selection algorithms

**Figure 4:**
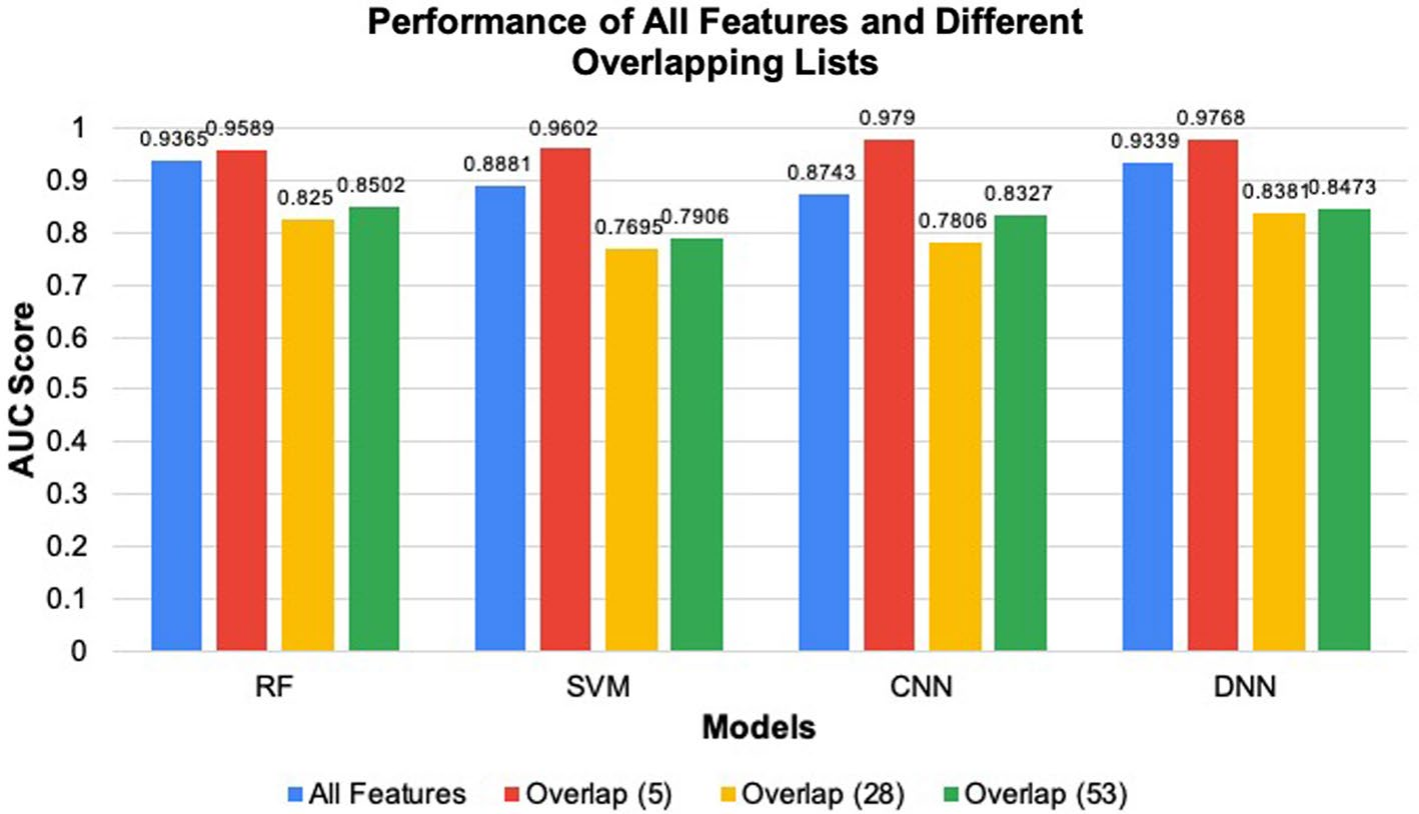
Comparing the AUC results of different genes lists. The blue bar is for the whole DEGs list (924 genes), the red bar is the overlapping list of LASSO and Ridge (5 genes). The yellow bar is the overlapping list of the six ranking algorithms (28 genes), and the green is the overlapping list of the ranking algorithms excluding MCC (53 genes).

As mentioned above, 28 hub genes were commonly identified through the six ranking algorithms, and when we exclude the genes identified by MCC, we found this list of commonly identified genes almost doubles to 53 hub genes. Thus, we also evaluated the prediction performances of these overlapping genes. We found that of the genes common to all the ranking algorithms (the 28 and 53 hub genes), the 53 hub genes achieved higher AUC for all the models than the 28 genes (Fig. 4). Moreover, with the 53 hub genes, the DNN and RF models achieved better prediction performances. Specifically, the DNN model achieved the best prediction performance with an AUC = 0.8473. Beyond this, the feature selection algorithms LASSO and Ridge commonly identified five genes, including FCER1G, PDE6H, SEMA6A, SLC25A46, and SST. When evaluating the prediction performances of the models with these five genes, we similarly found that deep learning models achieved better prediction performances than the machine learning models. Specifically, the CNN model achieved the best prediction performance with an AUC = 0.979. Moreover, the prediction performances of the models with these five genes achieved AUC’s ranging from 0.9589 to 0.979, which is surprising as the five hub genes commonly identified by the feature selection algorithms are not a subset of the 28 or 53 hub genes identified through the ranking algorithms.

These results suggest that even though several research groups built classifiers that achieved good prediction performances using hub genes^35,36^, we can likely improve the classifier’s prediction performances by using feature selection algorithms such as LASSO and Ridge instead. Also, the AUC = 0.979 achieved with the five genes (commonly identified by the LASSO and Ridge algorithms) is very close to the highest AUCs achieved with the 27 (AUC = 0.9896) and 41 (AUC = 0.9841) genes identified by the LASSO and Ridge algorithms, respectively. This finding is crucial as even though we have moved to a time where high throughput expression data is more easily attainable, medical facilities are still largely implementing the practice of only testing a small set of genes to determine the diagnosis. Thus, incorporating the five genes into current medical practice should be more readily accepted than long lists of genes. Nonetheless, more experiments are needed before this idea will be feasible.

#### Evaluating the performance of the classification models on independent sets

To further assess the robustness of our best models. We tested these models on two independent datasets (GSE109887 and GSE138260, see Table 1). We performed the external testing using two of the best-constructed models, the DNN model built by the 27 key genes selected by LASSO and the DNN model built using 41 key genes selected by Ridge.

In this experiment, we used all the samples of the combined dataset to train the DNN model and then tested the model using the external datasets. For the GSE109887 brain tissue dataset from the medial temporal gyrus, our best model successfully classifies the samples using the 41 genes, and achieves AUC = 0.8546, F1 = 0.7524, Recall = 0.7564, and Precision = 0.7549. Also, for the GSE138260 brain tissue dataset, our best model classified the samples with AUC = 0.7523, F1 = 0.7205, Recall = 0.7222, and Precision = 0.737. The results show that even though the cross-validation shows several feature sets give high accuracy, performing the external testing, we found that the best feature set is the 41 genes since it performed well in both the training and the independent testing sets.

### Bioinformatics analyses of the overlapping gene sets

**Figure 5:**
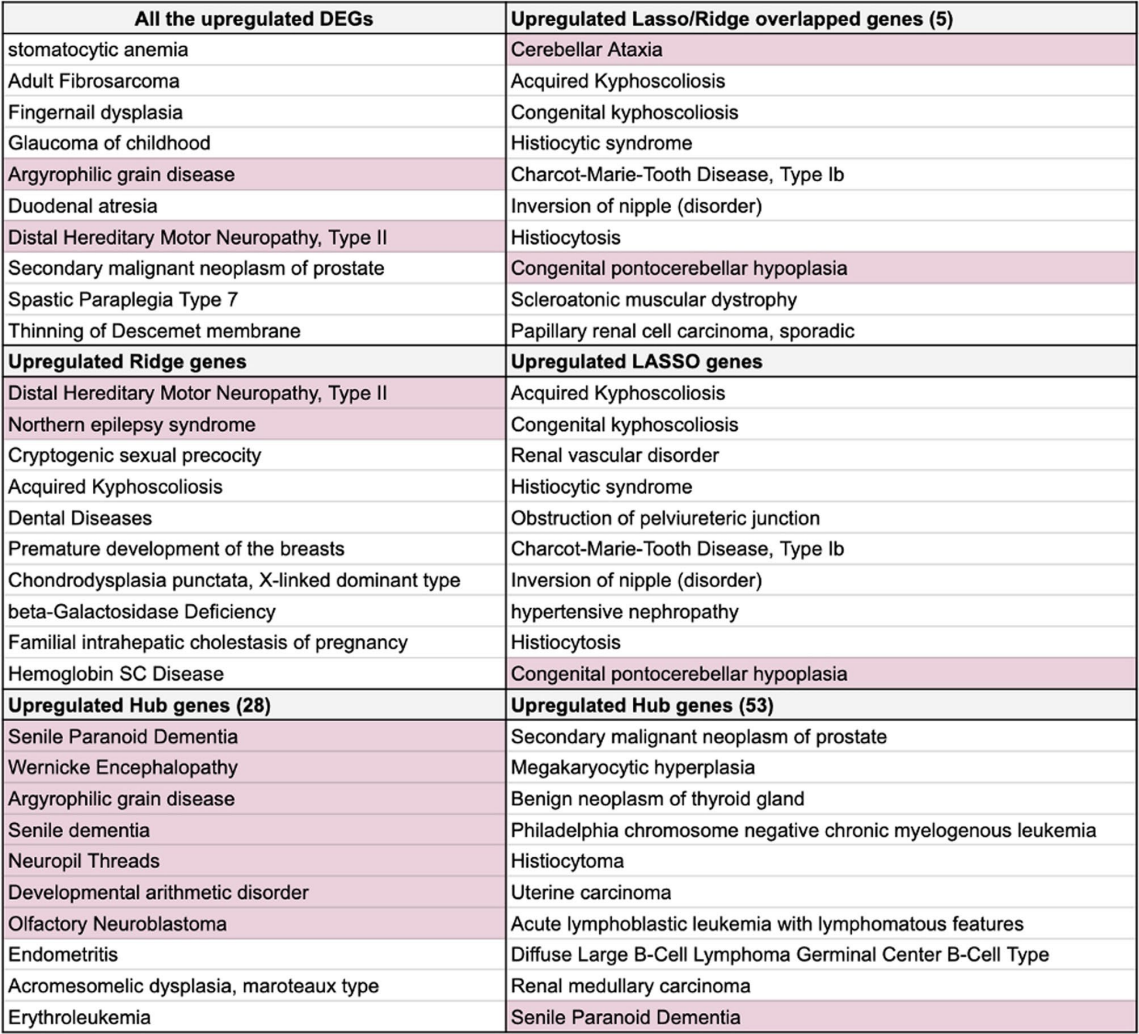
The top-10 enriched diseases retrieved from DisGeNET for upregulated genes in the different gene sets, including the DEGs, LASSO, Ridge, and the overlapped/common hub and feature selection genes.

To determine which set of overlapping genes is more aligned with the underlying biology of AD based on different bioinformatics tools, we used the comprehensive gene set analyses tool, Enrichr^32^. First, we determined which gene set is linked to the most neurological disorders using DisGeNET in Enrichr. For this process, we only used the upregulated genes that in essence contributors to the features that characterize AD. Figure 5 provides the top-10 enriched diseases retrieved from DisGeNET for all the overexpressed genes in the LASSO, Ridge, and DEGs gene set, as well as the overlapping gene sets. We then determined all the neurological disorders in each gene set (highlighted in pink). All the upregulated DEGs and all the overlapping gene sets, only had two or less neurological diseases/disorders featuring in the enriched top-10 diseases, except the 28 overlapping hub gene set. For the upregulated genes from the 28 overlapping hub genes, 7 of the top-10 enriched diseases were neurological diseases/disorders, suggesting that this list likely captures more of the underlying AD pathophysiology.

For the 5 overlapping genes identified through the feature selection methods (LASSO and Ridge), two of the potentially identified biomarkers SST and SEMA6A are also identified in Perera et al.^16^, Yu et al.^18^, and Madar et al.^15^. MT1M is also one of the genes selected by LASSO, the subset that achieved high accuracy in distinguishing AD and control samples, and was also identified as a potential biomarker in Perera et al.^16^. For the 28 overlapping genes identified through the ranking algorithms, one of the potentially identified biomarkers BDNF is also identified in Yu et al.^18^. This shows the overlap between our study and other studies^15–18^ specifically is very small.

We subsequently conducted a literature review to determine which of the ten upregulated hub genes (among the 28 overlapping hub genes) have already been recognized as AD therapeutic targets. There are currently many research efforts to provide disease-modifying therapies for AD treatment^37^. The key targets are Tau (the MAPT gene product) and amyloid precursor protein (APP), as they are the significant components of neurofibrillary tangles and amyloid plaques, respectively^38,39^. Brain-derived neurotrophic factor (BDNF) functions as a ligand for neurotrophic tyrosine kinase receptor type 2 (NTRK2). BDNF stimulates NTRK2 to phosphorylate APP, causing its accumulation in the TGN (Trans-Golgi Network), diminishing its amyloidogenic cleavage. However, BDNF is reduced in AD, while levels of APP increase. Moreover, NTRK2 and APP are cleaved by δ-secretase in AD brains, and blocking TrkB cleavage in 5xFAD mice attenuated AD pathologies^40^. Also, Aβ triggers PDZ-dependent recruitment of PTEN into the postsynaptic compartment to induce synaptic toxicity and cognitive dysfunction, which offers a new mechanism-based therapeutic target to counteract downstream Aβ signaling^41,42^. Thus, APP, NTRK2, PTEN, and the MAPT gene product (Tau) are all aggregate protein-related therapeutic targets.

Moreover, the JAK2 inhibitor, TG101209, attenuated the IFNγ-induced changes in cultured microglia and microglia from APP/PS1 mice^43^. Also, Raf inhibitor, sorafenib, reversed memory impairment and reduced the expression of APP, Cox-2, and iNOS in the brain of an AD transgenic mouse model, which also suggests targeting RAF1^44^. These works suggest JAK2 or RAF1 as AD targets and potential strategies for reducing AD’s neuroinflammation. George et al.^45^ also recently demonstrated that long-term suppression of (insulin-like growth factor 1 receptor) IGF1R signaling alleviates AD progression and promotes neuroprotection in animal models.

This shows that a substantial portion of the pinpointed upregulated hub genes (70%) are AD targets and suggests that JUN, CYCS, and PSMD4 should also be explored. This is in line with recent reviews^46–48^ that suggest inhibitors of the mitogen-activated protein kinases (MAPK) pathways, such as the c-Jun N-terminal kinase (JNK) pathway, be tested for AD treatment as JNK3 enhances Aβ production and plays a key role in the maturation and development of neurofibrillary tangles.

**Figure 6:**
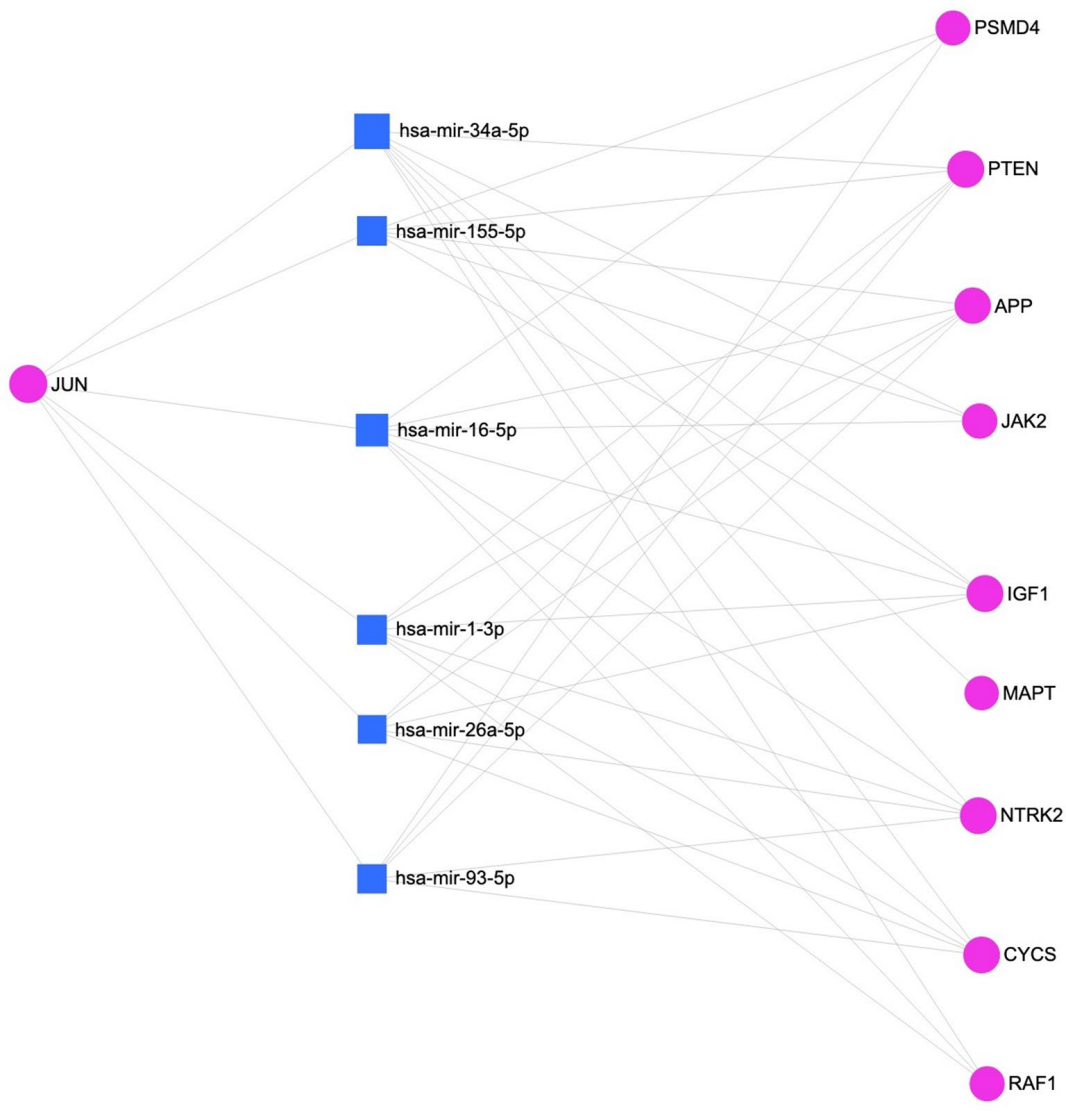
Network generated by miRNet. It shows six important miRNAs, represented by blue squares, and highlights JUN as the critical transcription factor.

To further determine the key set of microRNA and transcription factors associated with the upregulated genes among the 28 overlapping hub genes, we used miRNet^33^ with a network degree filter cutoff of 5.0. Figure 6 provides the network generated with miRNet that shows six important miRNA (hsa-mir-16-5p, hsa-mir-34a-5p, hsa-mir-1-3p, hsa-mir-26a-5p, hsa-mir-93-5p, hsa-mir-155-5p) and highlights JUN as the critical transcription factor participating in this process. Since 2020 four of the six microRNA were shown to be potential AD targets. Specifically, miR-16-5p^49^ and miR-34a-^50^ were shown to relieve amyloid β-induced injury by decreasing apoptosis and oxidative stress via targeting BACE1, and overexpression of miR-26a-5p suppresses Tau phosphorylation and Aβ accumulation^51^, indicating these microRNAs downregulated in AD are protective agents, and their increase can be targeted as a treatment. Moreover, another study show inhibitor of NF-κB kinase β (IKKβ) knockdown and miR-155-5p inhibition ameliorated cognitive impairment, improved neuron regeneration, and attenuated Aβ deposition in APP/PS1 mice, suggesting miR-155-5p as a target for AD treatment^52^.

Together these results suggest that even though the feature selection methods are better for the classification of samples, in this case, the 28 overlapping hub genes may be better for analyzing the underlying pathophysiology of AD and for pinpointing the potential targets, compared to the 5 overlapping genes identified through the feature selection methods. Additionally, the obtained results and follow-up investigation can deliver new insights into AD treatment.

## Concluding remarks

Decades of research have been poured into understanding the underlying pathophysiology of AD, yet we still do not have a cure for AD. And more recently, these efforts have been focused on identifying more potential targets for drug development as past drug development failures were found to be a result of inefficient targets. In parallel, advances associated with high throughput technology made more data available to build classifiers that can serve as decision support tools for clinicians. Thus, several research groups have built classifiers using hub genes and feature selection methods, and further used these gene sets to unveil more of the diseases’ underlying pathophysiology Thus, we here used both hub and feature selection genes to build ML/DL classifiers that can best distinguish AD samples from healthy aged controls. We also used the overlapping genes which allow zooming in on genes commonly pinpointed by more than one approach which increases confidence in their reliability. In our case, the five genes (commonly identified by the LASSO and Ridge algorithms) that produce a prediction performance (AUC = 0.979) similar to the best-performing method with 27 to 41 genes, can be incorporated into current medical practice more easily than a larger number of genes. Second, using only the overlapping hub genes and specifically, the upregulated ones makes the data less convoluted to show its link to the disease and the literature review further suggests that the majority of the upregulated overlapping hub genes may be targets, as well as the microRNAs that target multiple of the genes in this specific set.

## Data availability

In this study, we used publicly available gene expression datasets. These datasets can be found on Gene Expression Omnibus, *(accessed by April 2022),* https://www.ncbi.nlm.nih.gov/geo/. The source code of the ML\DL models in this work is available on: https://github.com/HindAlamro/AD_biomarker.

## Supplementary Information

Supplementary Information.

## References

[1] Alzheimer's A (2013). Alzheimer's disease facts and figures. Alzheimers. Dement..

[2] Alzheimer's A (2016). Alzheimer's disease facts and figures. Alzheimers. Dement..

[3] Long JM, Holtzman DM (2019). Alzheimer disease: An update on pathobiology and treatment strategies. Cell.

[4] Cummings JL, Morstorf T, Zhong K (2014). Alzheimer's disease drug-development pipeline: few candidates, frequent failures. Alzheimers. Res. Ther..

[5] Wang J, Gu BJ, Masters CL, Wang Y-J (2017). A systemic view of Alzheimer disease - insights from amyloid-β metabolism beyond the brain. Nat. Rev. Neurol..

[6] Bajic VP (2019). The X files: "the mystery of X chromosome instability in Alzheimer's disease". Front. Genet..

[7] Jo T, Nho K, Saykin AJ (2019). Deep learning in Alzheimer's Disease: diagnostic classification and prognostic prediction using neuroimaging data. Front. Aging Neurosci..

[8] Alamro H (2023). Type 2 diabetes mellitus and its comorbidity, Alzheimer’s disease: identifying critical microRNA using machine learning. Front. Endocrinol..

[9] Ludwig N (2019). Machine learning to detect Alzheimer's disease from circulating non-coding RNAs. Genomics Proteomics Bioinform..

[10] Qorri B, Tsay M, Agrawal A, Au R, Gracie J (2020). Using machine intelligence to uncover Alzheimer’s disease progression heterogeneity. Explor. Med..

[11] Xu A, Kouznetsova VL, Tsigelny IF (2022). Alzheimer's disease diagnostics using miRNA biomarkers and machine Learning. J. Alzheimers. Dis..

[12] Monk B (2021). A machine learning method to identify genetic variants potentially associated with Alzheimer's disease. Front. Genet..

[13] Rodriguez S (2021). Machine learning identifies candidates for drug repurposing in Alzheimer’s disease. Nat. Commun..

[14] Urbina F, Puhl AC, Ekins S (2021). Recent advances in drug repurposing using machine learning. Curr. Opin. Chem. Biol..

[15] Madar IH (2021). Identification of marker genes in Alzheimer's disease using a machine-learning model. Bioinformation.

[16] Perera, S. *et al.* In *2020 Moratuwa Engineering Research Conference (MERCon)* 1–6 (2020).

[17] Zhao X, Yao H, Li X (2021). Unearthing of Key genes driving the pathogenesis of Alzheimer's disease via bioinformatics. Front. Genet..

[18] Yu W, Yu W, Yang Y, Lü Y (2021). Exploring the key genes and identification of potential diagnosis biomarkers in Alzheimer's disease using bioinformatics analysis. Front. Aging Neurosci..

[19] Clough E, Barrett T (2016). Methods Molecul. Biol..

[20] Liang WS (2007). Gene expression profiles in anatomically and functionally distinct regions of the normal aged human brain. Physiol. Genomics.

[21] Berchtold NC (2008). Gene expression changes in the course of normal brain aging are sexually dimorphic. Proc. Natl. Acad. Sci. U. S. A..

[22] Blalock EM (2004). Incipient Alzheimer's disease: Microarray correlation analyses reveal major transcriptional and tumor suppressor responses. Proc. Natl. Acad. Sci. U. S. A..

[23] Lardenoije R (2019). Alzheimer's disease-associated (hydroxy)methylomic changes in the brain and blood. Clin. Epigenetics.

[24] Nitsche A (2021). Alzheimer-related genes show accelerated evolution. Mol. Psychiatry.

[25] Toro-Domínguez D (2019). ImaGEO: Integrative gene expression meta-analysis from GEO database. Bioinformatics.

[26] Gardener M (2012). Beginning R: The statistical programming language.

[27] Szklarczyk D (2020). The STRING database in 2021: Customizable protein–protein networks, and functional characterization of user-uploaded gene/measurement sets. Nucleic Acids Res..

[28] Kohl M, Wiese S, Warscheid B (2011). Cytoscape: Software for visualization and analysis of biological networks. Methods Mol. Biol..

[29] Chin C-H (2014). cytoHubba: Identifying hub objects and sub-networks from complex interactome. BMC Syst. Biol..

[30] Kim, Y. & Kim, J. Gradient LASSO for feature selection. In *Twenty-first international conference on Machine learning - ICML '04* (2004) 10.1145/1015330.1015364

[31] Zhang S, Cheng D, Hu R, Deng Z (2018). Supervised feature selection algorithm via discriminative ridge regression. World Wide Web.

[32] Kuleshov MV (2016). Enrichr: A comprehensive gene set enrichment analysis web server 2016 update. Nucleic Acids Res..

[33] Chang L, Zhou G, Soufan O, Xia J (2020). miRNet 2.0: Network-based visual analytics for miRNA functional analysis and systems biology. Nucleic Acids Res..

[34] Stoesser, G. NCBI (National center for biotechnology information). *Dictionary of bioinformatics and computational biology* (2004).10.1002/0471650129.dob0477

[35] Wee JJ, Kumar S (2020). Prediction of hub genes of Alzheimer's disease using a protein interaction network and functional enrichment analysis. Genomics Inform..

[36] Gui H, Gong Q, Jiang J, Liu M, Li H (2021). Identification of the hub Genes in Alzheimer's disease. Comput. Math. Methods Med..

[37] Cummings J, Lee G, Ritter A, Sabbagh M, Zhong K (2019). Alzheimer's disease drug development pipeline: 2019. Alzheimers. Dement..

[38] Soeda Y, Takashima A (2020). New insights into drug discovery targeting tau protein. Front. Mol. Neurosci..

[39] Zhao J, Liu X, Xia W, Zhang Y, Wang C (2020). Targeting amyloidogenic processing of APP in Alzheimer's disease. Front. Mol. Neurosci..

[40] Xia Y (2021). TrkB receptor cleavage by delta-secretase abolishes its phosphorylation of APP, aggravating Alzheimer's disease pathologies. Mol. Psychiatry.

[41] Giebink GS (1989). Progress in understanding the pathophysiology of otitis media. Pediatr. Rev..

[42] Frere S, Slutsky I (2016). Targeting PTEN interactions for Alzheimer's disease. Nat. Neurosci..

[43] Jones RS, Minogue AM, Fitzpatrick O, Lynch MA (2015). Inhibition of JAK2 attenuates the increase in inflammatory markers in microglia from APP/PS1 mice. Neurobiol. Aging.

[44] Burgess S, Echeverria V (2010). Raf inhibitors as therapeutic agents against neurodegenerative diseases. CNS Neurol. Disord. Drug Targets.

[45] George C (2017). The Alzheimer's disease transcriptome mimics the neuroprotective signature of IGF-1 receptor-deficient neurons. Brain.

[46] Yarza R, Vela S, Solas M, Ramirez MJ (2015). c-Jun N-terminal Kinase (JNK) signaling as a therapeutic target for Alzheimer's disease. Front. Pharmacol..

[47] Busquets O (2021). c-Jun N-terminal kinases in Alzheimer's disease: A Possible target for the modulation of the earliest alterations. J. Alzheimers. Dis..

[48] Okazawa H, Estus S (2002). The JNK/c-Jun cascade and Alzheimer's disease. Am. J. Alzheimers. Dis. Other Demen..

[49] Zhang N (2020). miR-16-5p and miR-19b-3p prevent amyloid β-induced injury by targeting BACE1 in SH-SY5Y cells. NeuroReport.

[50] Li P, Xu Y, Wang B, Huang J, Li Q (2020). miR-34a-5p and miR-125b-5p attenuate Aβ-induced neurotoxicity through targeting BACE1. J. Neurol. Sci..

[51] Liu Y (2020). Overexpression of miR-26a-5p suppresses tau phosphorylation and Aβ accumulation in the Alzheimer's disease mice by targeting DYRK1A. Curr. Neurovasc. Res..

[52] Wang W (2022). MicroRNA-155-5p targets SKP2, activates IKKβ, increases Aβ aggregation, and aggravates a mouse Alzheimer disease Model. J. Neuropathol. Exp. Neurol..

