# Supplementary material for: Exploiting machine learning models to identify novel Alzheimer’s disease biomarkers and potential targets
Source: Sci Rep. 2023 Mar 27;13:4979. doi: 10.1038/s41598-023-30904-5 (PMC10043000; doi:10.1038/s41598-023-30904-5)
Supplement: Supplementary file 1 — Supplementary Information. [file 41598_2023_30904_MOESM1_ESM.docx]

**Exploiting Machine Learning Models to Identify Novel Alzheimer’s Disease Biomarkers and Potential Targets**

**Supplementary Materials**

**Supplementary Table S1:** The complete list of the identified DEGs. In total they are 924 DEGs, 521 upregulated and 403 down-regulated A) shows upregulated DEGs, B) shows down-regulated DEGs

| 1. **Upregulated DEGs** | | | | | | | | |
| --- | --- | --- | --- | --- | --- | --- | --- | --- |
| TEX10 | TGFBR3 | LPAR4 | PAAF1 | | CBX3 | SEMA6A | FDFT1 | GUK1 |
| NSUN6 | P2RY6 | COL11A2 | COL1A1 | | TMF1 | PRPF18 | RABGAP1 | GYPE |
| KCNE4 | ZSWIM1 | SLC2A1 | TMED10 | | B4GALT2 | A4GALT | LETM1 | SLC20A1 |
| ANP32B | FOXO4 | PRKRA | ARHGAP44 | | HIF3A | GPR4 | SFXN1 | COIL |
| MT1M | RHBDD3 | TMEM106C | GNA12 | | S100A4 | USP48 | TNFRSF14 | XRCC2 |
| SLC25A46 | ADD1 | LCAT | PRDM12 | | SOX4 | CEP57 | ANKRD12 | RAI2 |
| CCNC | PRMT2 | XRCC6 | GADD45G | | ALDH4A1 | DDX24 | TCFL5 | TAF11 |
| NOTCH2NL | DDX56 | ACADS | GJA4 | | PTGIS | EFNA1 | UROD | MACF1 |
| SLC22A18AS | PFKFB3 | LPAR1 | PTEN | | HSPA12A | LSR | SIRT5 | ANG |
| CXCR4 | SLC38A7 | SUB1 | APLNR | | SDAD1 | PRKCDBP | RNF122 | ST6GALNAC2 |
| HSD17B7 | MT2A | MAP1B | PRDX6 | | SLC12A7 | LIMS2 | APOD | CDC42BPB |
| RUFY3 | PLXNB1 | ZNRF4 | WAS | | C1orf61 | EP300 | TXNL1 | IFITM1 |
| PLSCR4 | VEZF1 | PTMA | TNFAIP2 | | MXRA8 | CD248 | CAV1 | NDUFA2 |
| KIAA0100 | PSTPIP1 | SGSM2 | LTF | | PRMT1 | DSPP | NUPR1 | RIN2 |
| GLTSCR1 | NMB | TOB1 | TEX261 | | CFDP1 | ITGA10 | G0S2 | WDR11 |
| PIK3CB | MICALL2 | CBR4 | FAM98A | | HOXB5 | MYCN | LZTR1 | LRCH4 |
| LRP4 | RASL12 | POFUT1 | MLC1 | | EPB42 | FLRT1 | MED27 | CENPF |
| ANKRD28 | EMC10 | RUNX3 | AMOTL2 | | SENP3 | CDC25A | RAPGEF3 | ZNF551 |
| DDIT4 | PDLIM4 | MT1X | MAFF | | FAM189A2 | GP6 | DLGAP2 | SMAD1 |
| PDLIM1 | TCIRG1 | SPR | ABLIM1 | | PNMA3 | RPS21 | NRIP2 | PGAM2 |
| PXDC1 | MUC6 | HSPB8 | PML | | MAPT | SPG21 | FGR | NLGN3 |
| HLA-DRB4 | SERPINI2 | TAF15 | TRMT61A | | PRRG3 | FOXF1 | USP4 | TNFRSF1B |
| TIMM8A | PPM1F | CHD1 | GALE | | DAP3 | TPP1 | INHBB | ISG20 |
| CNTD2 | WDR82 | MT1H | LUZP1 | | IRF2BP1 | RPS19 | PALM | JUNB |
| AEBP1 | FOXD1 | ELF1 | SPI1 | | UBE2D4 | COPE | BHMT2 | PRSS23 |
| HEY2 | MYOM1 | ENTPD2 | RBM25 | | PHEX | PSMD5 | TSPAN31 | TRIP10 |
| TAOK2 | OSBPL11 | PTH1R | EXT1 | | ASIC1 | IL1RAPL2 | MICAL3 | TRIM33 |
| RAB13 | RNPEPL1 | DBR1 | MYL3 | | S100A12 | RPS6KB2 | PPP1R13L | FAM172A |
| PYCR1 | NCOR2 | PMEPA1 | FTSJ1 | | GLTP | TMEM40 | CHD4 | RDH11 |
| APPL1 | DVL1 | EMC3 | SENP5 | | SH2D3C | MERTK | KCND1 | SCAF11 |
| AFG3L2 | CXCL1 | NBAS | PTAFR | | FBXO24 | TMPRSS5 | INPPL1 | CSNK1D |
| PCDH12 | EFHD1 | CASP7 | BAG3 | | PAX6 | RGP1 | KIFC3 | C7orf43 |
| IL10RA | GOLGA8A | FAM162A | ALDH5A1 | | RAF1 | MPP2 | FOXJ1 | MICB |
| RNF41 | TRIL | SLCO3A1 | FOXC1 | | UBE2Z | MYO1C | ADRA2C | SFPQ |
| GRAMD1C | PPP1R16B | SECISBP2L | SCML2 | | ASIC4 | KIF1C | TRAF3IP2 | JUN |
| CDS2 | CDIP1 | MT1F | ESPN | | VAPA | HAP1 | ATP5E | FOXJ3 |
| GRAMD3 | ZCCHC24 | SIGLEC1 | RHBDF2 | | TSC2 | CARM1 | TTC31 | ZFP36 |
| ID3 | TBX6 | NXN | LRP10 | | HDAC2 | R3HDM1 | NRXN2 | TEFM |
| ACAD10 | MFHAS1 | ZNF500 | | KLHL21 | TOP1 | METTL22 | KIF22 | TGOLN2 |
| BAZ1A | MZT2B | CEBPB | | PCOLCE | PTMS | PRPF38B | DAZAP1 | CALCA |
| PPP1R3D | UMPS | MAGI2 | | PPIG | C22orf24 | TMEM184B | PARP6 | VAMP5 |
| NFATC4 | SOX9 | COL4A3BP | | CD3EAP | THBS2 | TTC27 | STOML1 | NMT2 |
| WDR12 | TLN1 | EVC | | COL21A1 | SERPINA5 | GEM | HIBCH | TBX19 |
| RSRC2 | PLA2G16 | DENND1C | | MED14 | CERS4 | EMX2 | NEU3 | MARK3 |
| LPGAT1 | CFHR2 | DSTYK | | ECE2 | ARTN | NFAT5 | CDH22 | MYRF |
| FXYD3 | PSMD4 | CYP4F12 | | TMEM47 | GPATCH8 | GRIK2 | TRIM38 | GJA1 |
| CDC14A | PDZD3 | RHOQ | | TCEB2 | C19orf54 | SULT1A1 | R3HDM4 | STX17 |
| CDK5RAP2 | SLC7A9 | PIP | | NAPG | APOL3 | ZNF274 | EMP3 | ZC3H15 |
| DGKB | EPB41 | LTK | | PAX5 | NECAP2 | SNX11 | KCTD17 | KIAA0040 |
| CDK5R2 | ACOX3 | COPS8 | | LCN2 | MAP1A | ATP1A2 | MRPL20 | MPZ |
| HSP90B1 | HIST1H1C | RALA | | MSX1 | NEFM | UBQLN4 | ADD3 | SOCS3 |
| ITFG2 | PARP4 | STAP2 | | SNTA1 | SYNC | CLDN15 | PRKDC | WWOX |
| VCAM1 | CSK | PODNL1 | | HIST1H2AC | PLXDC1 | NTRK2 | AMPD2 | ERAL1 |
| RARRES2 | PRR14L | GSDMD | | JAK2 | IL3RA | LAMB2 | MAPKAPK3 | HSPA2 |
| ZNF335 | FBXO38 | KLC2 | | DND1 | SH2D2A | SLC2A9 | BIRC7 | CSPG4 |
| MLPH | IGF1 | A2M | | 3-Mar | TGFB1I1 | STK24 | ALX4 | DALRD3 |
| CD3D | PTPN13 | RAB1B | | CPNE3 | FHOD1 | SKI | IL17RB | BCAM |
| ARHGAP5 | NKX2-2 | FUT6 | | SYT11 | VASP | KIAA0141 | DNMT3B | NDUFB1 |
| MFN2 | COPB2 | UBE2W | | APEX2 | PGLYRP4 | MKRN3 | WBP1L | TNFRSF1A |
| AKR1C3 | GUCA1B | MATN4 | | RTN4 | FMO2 | STAG1 | CLDN5 | ARRB1 |
| CLEC1A | IQGAP1 | VGLL4 | | HDAC5 | BARX1 | HAUS3 | CYP4A11 | PTRF |
| RAMP2 | ABL1 | S100A8 | | POU3F2 | RRP1 | SLC12A9 | HDGFRP3 | UTP18 |
| STAT5A | ZC2HC1C | WDR13 | | IRS4 | CLNS1A | AQP3 | LYL1 | ACSM5 |
| PI4K2A | SYNPO | CLDND1 | | ERF | EVL | RBM38 | CYCS | CYP4F2 |
| APP | LCMT2 | UCHL5 | | CNN2 | CNTRL |  |  |  |

| 1. **Down-regulated DEGs** | | | | | | | |
| --- | --- | --- | --- | --- | --- | --- | --- |
| HSD17B4 | THYN1 | FXYD7 | HCCS | IMMT | TPX2 | FADS3 | MRPL48 |
| ACO2 | KCNH1 | ADRA1B | RNF146 | DDX46 | DAD1 | NPY5R | UBE2D2 |
| SRP54 | CA10 | ZNF706 | PFN2 | SPOCK3 | DYNLT3 | CTSB | TASP1 |
| PCDH8 | SERPINF1 | RLF | GLRX | TUBB4B | COG7 | ZMYND11 | MTHFD1 |
| ECHDC1 | ZNF665 | CITED1 | SH3GLB1 | STYK1 | MAGED2 | IPO7 | ZBBX |
| ZWINT | CDH13 | CDC7 | PHF14 | MEF2C | C14orf2 | RASA1 | TMBIM4 |
| TRO | TMEM168 | ANKRD11 | ATOX1 | SIAH2 | MKKS | PARP2 | NT5M |
| AMD1 | ZNF12 | RGS4 | PPCS | ACTR10 | NHP2 | TMEM14A | NEUROD6 |
| HNRNPL | GPR63 | CAMK1G | GSTA4 | PUM1 | HMP19 | GLIPR1 | EDC3 |
| THY1 | BTN3A3 | IARS | SLC30A3 | RNF111 | CUTC | UNC13B | NUDT9 |
| MRPL40 | KCNA4 | ORC3 | E2F5 | LAGE3 | MDH2 | ATXN10 | PPP1R7 |
| SEC31A | C2orf42 | KCNK7 | CCNH | CRHBP | ITPR1 | NRN1 | NAP1L1 |
| DHRS7 | RER1 | CTPS1 | PPP3CA | CORO2B | COPS5 | FABP3 | NMU |
| ATP1A1 | MMP25 | FKBP1B | ZC3H14 | MAPK10 | SAP130 | FCER1G | GARS |
| PRR11 | MRPS28 | FBXO34 | NDUFAB1 | MAP3K12 | DHRS7B | COPS7A | ETFDH |
| SCG5 | ATP5C1 | MRPL9 | CISD1 | SNRPC | MAGED1 | DECR1 | NOP10 |
| AP2A2 | UBE2N | GABRG3 | GNG3 | CHMP5 | ARC | MFF | RPL24 |
| NDUFV2 | ACTR3B | GSTT2 | PRDX1 | DNASE1L2 | RABGGTB | PRKCZ | RRAGB |
| LAMB1 | FAM49A | REEP1 | GLRB | MOB4 | STMN2 | KARS | SPOCK1 |
| MAP1LC3B | RBFOX1 | GLA | UBC | QSOX1 | CREG1 | MKRN1 | PJA1 |
| ITPKA | HNRNPA0 | GABARAPL2 | NDUFA9 | CDK7 | CAPRIN2 | VSNL1 | OPN3 |
| CACNG3 | PNMAL1 | RPH3A | HMGN2 | MMADHC | IL27RA | TMEM246 | FBXL2 |
| APOO | LRP2BP | KPNA2 | SUCLA2 | BEX4 | PDCD6IP | C11orf24 | ANKRD6 |
| PRMT7 | PDE6H | CORO1C | SUPV3L1 | ACTL6B | HSPB3 | RPA3 | CX3CL1 |
| TUBA1C | USP39 | CYFIP2 | GNB5 | FSD1 | NIF3L1 | ATP6V0B | EPCAM |
| PEX11B | GPRASP1 | RPL6 | ALAS1 | RAP2C | MDH1 | SARS | RAB27B |
| USP8 | EIF2S1 | UQCRC1 | SLC9A6 | INSIG2 | AP2S1 | MFSD9 | NARS |
| ANKRD49 | SNRPA | METTL3 | RBMS1 | BSN | NRGN | TSR1 | C1orf216 |
| HRG | CDC37L1 | BMPR2 | FAM20B | CCT7 | EXTL2 | SLC25A3 | SLIT2 |
| HLF | PSMD7 | TUBA4A | PAK3 | DZIP3 | SCN3B | SLIT3 | ATP6V1A |
| OSBPL9 | DHCR24 | RIT2 | CRMP1 | SLC25A4 | TALDO1 | STAT4 | MRPL15 |
| MTX2 | PNN | CAP1 | TM9SF2 | RTN1 | SCG2 | MTPAP | PTPN3 |
| SGCE | UBE2V2 | TUBB3 | ENO2 | KBTBD11 | CMAS | UBL3 | FGF12 |
| RWDD2B | XPC | EPB41L3 | EIF3G | MPP1 | KIZ | CALB1 | EGR2 |
| UNC13A | ZMAT4 | SNAP91 | SULT4A1 | GABBR2 | CCK | ATP5B | UCHL1 |
| RAD51C | MAP2K4 | HEXB | DCLK1 | CARTPT | CD200 | UQCRFS1 | OSTF1 |
| LAMTOR3 | DYRK4 | NXT1 | LAMP5 | FHL2 | C3orf14 | SST | CD2BP2 |
| CDC40 | BDNF | FAU | CCNA1 | INA | GOT1 | EEF1A2 | PCYOX1L |
| MCCC1 | COX5B | CDH10 | MTSS1 | P4HTM | UQCRC2 | GUCY1B3 | GOT2 |
| BRAP | MICAL2 | PFDN5 | RRAGA | LMO4 | ZNF415 | FIG4 | AMPH |
| SF3B1 | FAM216A | SORL1 | GTF3C1 | MRPL46 | UGP2 | SNCA | IP6K2 |
| MAP2K1 | HARS2 | KCNF1 | ATP6V1G2 | PSMG2 | PSMB7 | CDC42 | RAB15 |
| ATP6V1B2 | SPINT2 | OSBPL3 | PPP1CB | PKM | CALM1 | NCOA4 | IST1 |
| SLC35B1 | RPS7 | TAGLN3 | ARGLU1 | ASCC3 | SNX10 | NRIP3 | DHX16 |
| PEBP1 | POLR2C | ADK | CRYM | NDRG4 | MOCS2 | NDN | SF3B5 |
| NSG1 | UBE2L3 | SERPINI1 | MRPS17 | SNX3 | NME1 | PGM3 | SNAP25 |
| GSTM3 | CHGB | CDC37 | HTR2A | RPL19 | MEAF6 | KIFAP3 | AARS |
| DUSP6 | USP9Y | GYG1 | TSC1 | ATPIF1 | WDR37 | NSA2 | MADD |
| BCAS2 | PSME1 | ACTR1B | MLLT11 | SEPHS1 | PSMD12 | RGS2 | DNTTIP2 |
| ZNF34 | COPS3 | NDUFA4 | PNOC | EPM2AIP1 | EIF2B3 | NPTX2 | SIRT3 |
| RWDD2A | STAR | PPA1 |  |  |  |  |  |

**Supplementary Table S2:** Lists of the top-100 genes by the ranking algorithms.

| 1. **List of BC Ranking Algorithm** | | | | | | | | | | | |
| --- | --- | --- | --- | --- | --- | --- | --- | --- | --- | --- | --- |
| **Rank** | **Name** | **BC Score** | **Rank** | **Name** | **BC Score** | **Rank** | **Name** | **BC Score** | **Rank** | **Name** | **BC Score** |
| 1 | PTEN | 48630.89976 | 26 | RBFOX1 | 9238.511692 | 51 | PPA1 | 6424.777019 | 76 | JAK2 | 5287.101219 |
| 2 | CDC42 | 45966.05768 | 27 | RAF1 | 9103.143463 | 52 | COPS5 | 6416.712029 | 77 | WDR37 | 5249.374198 |
| 3 | UBC | 42825.38402 | 28 | ARRB1 | 9005.155214 | 53 | WDR12 | 6367.729229 | 78 | CDK7 | 5201.739539 |
| 4 | EP300 | 42604.45535 | 29 | PEBP1 | 8585.19623 | 54 | CHD4 | 6216.047818 | 79 | CDC40 | 5195.0345 |
| 5 | JUN | 40847.5977 | 30 | NTRK2 | 8538.627295 | 55 | ATP5C1 | 6210.496982 | 80 | IQGAP1 | 5114.307171 |
| 6 | MAPT | 32227.86863 | 31 | ACO2 | 8165.375076 | 56 | KPNA2 | 6169.460823 | 81 | IMMT | 5038.59461 |
| 7 | SNAP25 | 29343.61077 | 32 | ATP6V1A | 8097.542966 | 57 | THY1 | 6109.705228 | 82 | TNFRSF1A | 5031.250979 |
| 8 | CAV1 | 25164.12218 | 33 | PSMD7 | 8013.113943 | 58 | EPB41 | 6081.930123 | 83 | PUM1 | 4883.511781 |
| 9 | CYCS | 24858.58663 | 34 | MAP2K1 | 7965.162092 | 59 | CXCR4 | 6010.411748 | 84 | PRKDC | 4879.055782 |
| 10 | APP | 24697.80717 | 35 | POLR2C | 7915.928061 | 60 | ADK | 5935.954913 | 85 | NDUFA4 | 4841.214557 |
| 11 | SNCA | 19967.20429 | 36 | XRCC6 | 7831.588751 | 61 | HIST1H2AC | 5872.478923 | 86 | AMPH | 4733.354712 |
| 12 | BDNF | 18247.38333 | 37 | COL1A1 | 7814.057136 | 62 | MEF2C | 5842.704322 | 87 | TLN1 | 4727.11799 |
| 13 | CCT7 | 16110.63309 | 38 | HDAC2 | 7678.243365 | 63 | TUBA4A | 5837.006849 | 88 | PI4K2A | 4708.12499 |
| 14 | SOX9 | 12583.50054 | 39 | MFN2 | 7630.372016 | 64 | PPP1CB | 5835.700712 | 89 | DDX24 | 4690.979079 |
| 15 | IGF1 | 12112.50443 | 40 | ABL1 | 7567.373188 | 65 | TAGLN3 | 5661.918708 | 90 | GOT2 | 4649.792411 |
| 16 | PSMD4 | 11966.34411 | 41 | ATP5B | 7339.425672 | 66 | TOP1 | 5647.873842 | 91 | CUX1 | 4645.171992 |
| 17 | VAPA | 11746.05036 | 42 | NME1 | 7211.495308 | 67 | CASP7 | 5585.805969 | 92 | PRKCZ | 4621.438211 |
| 18 | EIF2S1 | 11018.7827 | 43 | PPIG | 7024.67496 | 68 | HSP90B1 | 5481.491891 | 93 | GJA1 | 4553.443554 |
| 19 | CALM1 | 10938.86915 | 44 | MYO1C | 6926.606403 | 69 | SF3B1 | 5451.753091 | 94 | GNG3 | 4534.830304 |
| 20 | PKM | 10886.03023 | 45 | ENO2 | 6867.609738 | 70 | APOD | 5440.589811 | 95 | A2M | 4492.908041 |
| 21 | GNB5 | 10622.61123 | 46 | MT2A | 6859.799725 | 71 | RPS7 | 5405.334289 | 96 | WDR82 | 4477.609231 |
| 22 | CARM1 | 10370.71053 | 47 | STAT5A | 6731.266595 | 72 | MAPK10 | 5368.712832 | 97 | DNMT3B | 4465.51093 |
| 23 | ITPR1 | 10204.41242 | 48 | DVL1 | 6549.411411 | 73 | CSNK1D | 5336.406733 | 98 | GABARAPL2 | 4406.190801 |
| 24 | NHP2 | 10076.84609 | 49 | ATOX1 | 6546.013608 | 74 | RBM25 | 5306.618963 | 99 | HSD17B4 | 4405.016872 |
| 25 | PRMT1 | 10037.98043 | 50 | KARS | 6429.979425 | 75 | PFDN5 | 5303.636238 | 100 | FAU | 4378.084386 |

| 1. **List of Degree Ranking Algorithm** | | | | | | | | | | | |
| --- | --- | --- | --- | --- | --- | --- | --- | --- | --- | --- | --- |
| **Rank** | **Name** | **Dgr Score** | **Rank** | **Name** | **Dgr Score** | **Rank** | **Name** | **Dgr Score** | **Rank** | **Name** | **Dgr Score** |
| 1 | PTEN | 94 | 26 | RAF1 | 35 | 50 | UBE2V2 | 29 | 75 | CDK7 | 26 |
| 2 | JUN | 82 | 26 | ABL1 | 35 | 50 | STAT5A | 29 | 75 | TSR1 | 26 |
| 2 | UBC | 82 | 26 | XRCC6 | 35 | 50 | NDUFA4 | 29 | 75 | GNB5 | 26 |
| 4 | EP300 | 79 | 26 | NDUFAB1 | 35 | 50 | PEBP1 | 29 | 75 | PPA1 | 26 |
| 5 | CDC42 | 78 | 30 | MDH1 | 34 | 50 | UQCRC2 | 29 | 75 | STMN2 | 26 |
| 6 | MAPT | 65 | 31 | ENO2 | 33 | 50 | UQCRFS1 | 29 | 81 | CDC25A | 25 |
| 6 | SNAP25 | 65 | 31 | WDR12 | 33 | 50 | SOX9 | 29 | 81 | IMMT | 25 |
| 8 | CYCS | 59 | 31 | RPS7 | 33 | 50 | ARRB1 | 29 | 81 | GJA1 | 25 |
| 9 | SNCA | 58 | 31 | RPL6 | 33 | 50 | ITPR1 | 29 | 81 | PRMT1 | 25 |
| 10 | BDNF | 56 | 35 | MDH2 | 32 | 60 | MAPK10 | 28 | 85 | UCHL5 | 24 |
| 11 | APP | 55 | 35 | CXCR4 | 32 | 60 | HSP90B1 | 28 | 85 | RBM25 | 24 |
| 12 | CCT7 | 48 | 37 | MFN2 | 31 | 60 | SF3B1 | 28 | 85 | PIK3CB | 24 |
| 13 | IGF1 | 47 | 37 | JAK2 | 31 | 60 | UTP18 | 28 | 85 | SDAD1 | 24 |
| 14 | CAV1 | 46 | 37 | PFDN5 | 31 | 60 | RPL19 | 28 | 85 | IARS | 24 |
| 15 | ACO2 | 45 | 37 | RPS19 | 31 | 60 | UBE2N | 28 | 85 | WAS | 24 |
| 16 | NHP2 | 44 | 37 | MRPL15 | 31 | 66 | HIST1H2AC | 27 | 85 | SIRT3 | 24 |
| 17 | ATP5B | 43 | 37 | NDUFA9 | 31 | 66 | IQGAP1 | 27 | 85 | GOT2 | 24 |
| 18 | EIF2S1 | 42 | 37 | COPS5 | 31 | 66 | KARS | 27 | 85 | DDX56 | 24 |
| 19 | ATP5C1 | 39 | 44 | FAU | 30 | 66 | PRKDC | 27 | 85 | DDX24 | 24 |
| 20 | NTRK2 | 38 | 44 | POLR2C | 30 | 66 | RPS21 | 27 | 85 | VAPA | 24 |
| 20 | PSMD4 | 38 | 44 | UQCRC1 | 30 | 66 | SLC25A3 | 27 | 85 | MAP1B | 24 |
| 22 | MAP2K1 | 37 | 44 | PSMD12 | 30 | 66 | NME1 | 27 | 97 | CTPS1 | 23 |
| 22 | PSMD7 | 37 | 44 | CHD4 | 30 | 66 | NSA2 | 27 | 97 | ATP5E | 23 |
| 22 | PKM | 37 | 44 | CALM1 | 30 | 66 | SPI1 | 27 | 97 | SOCS3 | 23 |
| 22 | HDAC2 | 37 | 50 | EIF3G | 29 | 75 | ATP6V1A | 26 | 97 | TOP1 | 23 |

| 1. **List of Closeness Ranking Algorithm** | | | | | | | | | | | |
| --- | --- | --- | --- | --- | --- | --- | --- | --- | --- | --- | --- |
| **Rank** | **Name** | **Close Score** | **Rank** | **Name** | **Close Score** | **Rank** | **Name** | **Close Score** | **Rank** | **Name** | **Close Score** |
| 1 | PTEN | 421.233333 | 26 | ENO2 | 351.65 | 51 | TOP1 | 338.233333 | 76 | CDK7 | 331.7 |
| 2 | JUN | 413.666667 | 27 | HSP90B1 | 351.083333 | 52 | ATP5C1 | 338.166667 | 77 | MDH1 | 331.633333 |
| 3 | CDC42 | 404 | 28 | NME1 | 350.45 | 53 | GJA1 | 337.95 | 78 | FAU | 331.4 |
| 4 | UBC | 402.933333 | 29 | PRKDC | 349.316667 | 54 | TSC2 | 336.416667 | 79 | RPS19 | 331.333333 |
| 5 | EP300 | 401.816667 | 30 | ARRB1 | 348.583333 | 55 | EEF1A2 | 336.383333 | 80 | RPS21 | 331.166667 |
| 6 | MAPT | 398.333333 | 31 | SIRT3 | 347.783333 | 56 | CSK | 336.35 | 81 | NDUFA9 | 330.983333 |
| 7 | SNCA | 391.05 | 32 | MFN2 | 346.733333 | 57 | PPP1CB | 336.283333 | 82 | SFPQ | 330.616667 |
| 8 | CYCS | 386.15 | 33 | COPS5 | 345.883333 | 58 | TUBA4A | 335.716667 | 83 | SLC2A1 | 330.25 |
| 9 | APP | 383.083333 | 34 | MAP1LC3B | 345.45 | 59 | CHD4 | 335.433333 | 84 | TAGLN3 | 330.133333 |
| 10 | CAV1 | 380.683333 | 35 | XRCC6 | 345.433333 | 60 | DVL1 | 335.333333 | 85 | GOT2 | 330.116667 |
| 11 | SNAP25 | 380.1 | 36 | CALM1 | 345.233333 | 61 | EGR2 | 334.766667 | 86 | PIK3CB | 330.05 |
| 12 | BDNF | 376.633333 | 37 | UCHL1 | 345.033333 | 62 | TNFRSF1A | 334.75 | 87 | CEBPB | 329.983333 |
| 13 | MAP2K1 | 371.5 | 38 | PSMD12 | 344.966667 | 63 | SOX9 | 334.65 | 88 | KPNA2 | 329.816667 |
| 14 | IGF1 | 369.133333 | 39 | CXCR4 | 344.183333 | 64 | STAT5A | 334.583333 | 89 | CASP7 | 329.8 |
| 15 | EIF2S1 | 364.333333 | 40 | PRKCZ | 343.833333 | 65 | CDC25A | 334.45 | 90 | NDUFA4 | 329.533333 |
| 16 | ABL1 | 361.8 | 41 | MAPK10 | 343.333333 | 66 | PEBP1 | 334.3 | 91 | GABARAPL2 | 329.483333 |
| 17 | CCT7 | 357.133333 | 42 | ACO2 | 343.133333 | 67 | RPL6 | 334.283333 | 92 | PPP3CA | 329.433333 |
| 18 | NTRK2 | 357.133333 | 43 | IQGAP1 | 342.166667 | 68 | RPS7 | 334.133333 | 93 | RPL19 | 329.2 |
| 19 | ITPR1 | 356.883333 | 44 | POLR2C | 341.95 | 69 | PSMB7 | 334.016667 | 94 | EIF3G | 329.133333 |
| 20 | PSMD4 | 356.666667 | 45 | HIST1H2AC | 341.633333 | 70 | NDUFAB1 | 333.716667 | 95 | MAP2K4 | 328.9 |
| 21 | ATP5B | 355.483333 | 46 | JAK2 | 340.85 | 71 | NCOR2 | 333.683333 | 96 | HDAC5 | 328.716667 |
| 22 | RAF1 | 355.15 | 47 | KARS | 338.616667 | 72 | VAPA | 333.066667 | 97 | UBE2V2 | 328.183333 |
| 23 | PSMD7 | 355.083333 | 48 | TUBB3 | 338.533333 | 73 | GNB5 | 333.066667 | 98 | STMN2 | 327.7 |
| 24 | PKM | 353.866667 | 49 | SIRT5 | 338.516667 | 74 | PRMT1 | 332.516667 | 99 | HSPB3 | 327.533333 |
| 25 | HDAC2 | 352.533333 | 50 | NHP2 | 338.433333 | 75 | PRDX1 | 332.216667 | 100 | PML | 327.383333 |

| 1. **List of MCC Ranking Algorithm** | | | | | | | | | | | |
| --- | --- | --- | --- | --- | --- | --- | --- | --- | --- | --- | --- |
| **Rank** | **Name** | **MCC Score** | **Rank** | **Name** | **MCC Score** | **Rank** | **Name** | **MCC Score** | **Rank** | **Name** | **MCC Score** |
| 1 | UQCRFS1 | 2.02E+09 | 26 | FAU | 4.38E+06 | 51 | PSMD5 | 85928 | 76 | ITFG2 | 40321 |
| 2 | ATP5B | 2.02E+09 | 27 | EIF3G | 4.36E+06 | 52 | ATP6V1A | 85826 | 76 | RPMS17 | 40321 |
| 3 | ATP5C1 | 2.02E+09 | 28 | EIF2S1 | 4.01E+06 | 53 | PSME1 | 85704 | 78 | SNAP25 | 36607 |
| 4 | UQCRC2 | 2.02E+09 | 29 | PFDN5 | 4.00E+06 | 54 | TCIRG1 | 85692 | 79 | MAPT | 33828 |
| 5 | UQCRC1 | 2.02E+09 | 30 | ATP5E | 3.68E+06 | 55 | ATP6V1G2 | 85684 | 80 | BDNF | 33294 |
| 6 | MDH1 | 2.01E+09 | 31 | NSA2 | 1.87E+06 | 56 | ATP6V0B | 85680 | 81 | UCHL1 | 29576 |
| 7 | MDH2 | 2.01E+09 | 32 | IMMT | 1.46E+06 | 57 | MRPL46 | 81712 | 82 | MAP1B | 27801 |
| 8 | COX5B | 2.01E+09 | 33 | WDR12 | 1.19E+06 | 58 | DAP3 | 81703 | 83 | NTRK2 | 27706 |
| 9 | CYCS | 2.00E+09 | 34 | UTP18 | 1.18E+06 | 59 | MRPL40 | 81625 | 84 | SNCA | 24499 |
| 10 | NDUFV2 | 1.93E+09 | 35 | TSR1 | 1.18E+06 | 60 | MRPL20 | 80814 | 85 | APP | 20361 |
| 11 | NDUFA9 | 1.92E+09 | 36 | SDAD1 | 1.18E+06 | 61 | ATP6V1B2 | 80772 | 86 | STMN2 | 16408 |
| 12 | SLC25A3 | 1.06E+09 | 37 | RRP1 | 1.18E+06 | 62 | MRPL48 | 80742 | 87 | JUN | 15841 |
| 13 | NDUFA4 | 1.02E+09 | 38 | DDX56 | 1.14E+06 | 63 | MRPL9 | 80696 | 88 | EEF1A2 | 15293 |
| 14 | ACO2 | 1.01E+09 | 39 | DDX24 | 7.38E+05 | 64 | MRPS28 | 80690 | 89 | TUBB3 | 12740 |
| 15 | NDUFAB1 | 9.66E+08 | 40 | TEX10 | 7.32E+05 | 65 | APOO | 46246 | 90 | IGF1 | 12289 |
| 16 | SLC25A4 | 8.78E+07 | 41 | MRPL15 | 4.51E+05 | 66 | NDUFB1 | 45366 | 91 | MAP2K1 | 11112 |
| 17 | SUCLA2 | 1.49E+07 | 42 | NSUN6 | 4.49E+05 | 67 | GOT2 | 44907 | 92 | RAF1 | 10805 |
| 18 | NDUFA2 | 1.09E+07 | 43 | CCT7 | 3.72E+05 | 68 | CTPS1 | 42114 | 93 | CDC42 | 10233 |
| 19 | NHP2 | 5.90E+06 | 44 | DNTTIP2 | 3.64E+05 | 69 | PPA1 | 40607 | 94 | SRP54 | 10160 |
| 20 | RPS7 | 4.76E+06 | 45 | UBC | 1.07E+05 | 70 | RUNX3 | 40534 | 95 | UCHL5 | 8515 |
| 21 | RPS19 | 4.76E+06 | 46 | PTEN | 9.83E+04 | 71 | CDC25A | 40528 | 96 | ENO2 | 7425 |
| 22 | RPL6 | 4.76E+06 | 47 | PSMD4 | 9.38E+04 | 72 | RRAGA | 40502 | 97 | JAK2 | 7191 |
| 23 | RPS21 | 4.75E+06 | 48 | PSMD7 | 9.31E+04 | 73 | RRAGB | 40492 | 98 | CSK | 6089 |
| 24 | RPL19 | 4.75E+06 | 49 | PSMD12 | 9.12E+04 | 74 | FTSJ1 | 40440 | 99 | PEBP1 | 6066 |
| 25 | RPL24 | 4.38E+06 | 50 | PSMB7 | 9.07E+04 | 75 | LAMTOR3 | 40352 | 100 | NEFM | 5990 |

| 1. **List of MNC Ranking Algorithm** | | | | | | | | | | | |
| --- | --- | --- | --- | --- | --- | --- | --- | --- | --- | --- | --- |
| **Rank** | **Name** | **MNC Score** | **Rank** | **Name** | **MNC Score** | **Rank** | **Name** | **MNC Score** | **Rank** | **Name** | **MNC Score** |
| 1 | PTEN | 91 | 25 | ABL1 | 34 | 51 | MAPK10 | 27 | 75 | SDAD1 | 23 |
| 2 | JUN | 79 | 25 | NDUFAB1 | 34 | 51 | SF3B1 | 27 | 75 | KARS | 23 |
| 2 | UBC | 79 | 25 | HDAC2 | 34 | 51 | RPL19 | 27 | 75 | CTPS1 | 23 |
| 4 | EP300 | 77 | 29 | ENO2 | 32 | 51 | UBE2V2 | 27 | 75 | ATP5E | 23 |
| 5 | CDC42 | 75 | 29 | XRCC6 | 32 | 51 | STAT5A | 27 | 75 | WAS | 23 |
| 6 | SNAP25 | 62 | 29 | MDH1 | 32 | 51 | COPS5 | 27 | 75 | COX5B | 23 |
| 7 | MAPT | 61 | 32 | MFN2 | 31 | 51 | ITPR1 | 27 | 75 | GOT2 | 23 |
| 8 | CYCS | 58 | 32 | WDR12 | 31 | 58 | EIF3G | 26 | 75 | DDX56 | 23 |
| 9 | BDNF | 56 | 32 | RPL6 | 31 | 58 | PRKDC | 26 | 84 | EGR2 | 22 |
| 10 | SNCA | 55 | 32 | MRPL15 | 31 | 58 | RPS21 | 26 | 84 | HIST1H2AC | 22 |
| 11 | APP | 48 | 32 | CXCR4 | 31 | 58 | UQCRFS1 | 26 | 84 | PSMB7 | 22 |
| 12 | IGF1 | 46 | 37 | PSMD12 | 30 | 58 | STMN2 | 26 | 84 | SIRT3 | 22 |
| 13 | ATP5B | 43 | 37 | RPS7 | 30 | 58 | SOX9 | 26 | 84 | SLC25A3 | 22 |
| 14 | ACO2 | 42 | 37 | JAK2 | 30 | 58 | CALM1 | 26 | 84 | NDUFV2 | 22 |
| 14 | NHP2 | 42 | 37 | RPS19 | 30 | 58 | ARRB1 | 26 | 84 | SNAP91 | 22 |
| 16 | CCT7 | 41 | 37 | NDUFA9 | 30 | 66 | IQGAP1 | 25 | 84 | GJA1 | 22 |
| 16 | EIF2S1 | 41 | 37 | MDH2 | 30 | 66 | CDK7 | 25 | 84 | GNG3 | 22 |
| 18 | CAV1 | 39 | 43 | UQCRC1 | 29 | 66 | CDC25A | 25 | 84 | TUBB3 | 22 |
| 19 | ATP5C1 | 37 | 43 | UQCRC2 | 29 | 66 | UTP18 | 25 | 84 | DDX24 | 22 |
| 20 | NTRK2 | 36 | 45 | FAU | 28 | 66 | NDUFA4 | 25 | 84 | TSC2 | 22 |
| 20 | PKM | 36 | 45 | HSP90B1 | 28 | 66 | NME1 | 25 | 84 | UCHL1 | 22 |
| 22 | MAP2K1 | 35 | 45 | POLR2C | 28 | 66 | NSA2 | 25 | 97 | UCHL5 | 21 |
| 22 | PSMD7 | 35 | 45 | PFDN5 | 28 | 73 | TSR1 | 24 | 97 | CSK | 21 |
| 22 | PSMD4 | 35 | 45 | CHD4 | 28 | 73 | SPI1 | 24 | 97 | PIK3CB | 21 |
| 25 | RAF1 | 34 | 45 | UBE2N | 28 | 75 | RBM25 | 23 | 97 | IARS | 21 |

| 1. **List of Stress Ranking Algorithm** | | | | | | | | | | | |
| --- | --- | --- | --- | --- | --- | --- | --- | --- | --- | --- | --- |
| **Rank** | **Name** | **Stress Score** | **Rank** | **Name** | **Stress Score** | **Rank** | **Name** | **Stress Score** | **Rank** | **Name** | **Stress Score** |
| 1 | PTEN | 468334 | 26 | GNB5 | 86606 | 51 | JAK2 | 61582 | 76 | SIRT3 | 49604 |
| 2 | UBC | 403550 | 27 | PSMD7 | 84290 | 52 | PRKDC | 60938 | 77 | GOT2 | 49058 |
| 3 | CDC42 | 382894 | 28 | PRMT1 | 83662 | 53 | CHD4 | 60424 | 78 | MAPK10 | 49028 |
| 4 | EP300 | 379498 | 29 | VAPA | 83352 | 54 | HSP90B1 | 57886 | 79 | GJA1 | 48612 |
| 5 | JUN | 377914 | 30 | ABL1 | 83140 | 55 | NDUFAB1 | 56464 | 80 | DVL1 | 48576 |
| 6 | MAPT | 302920 | 31 | PEBP1 | 81488 | 56 | PFDN5 | 55708 | 81 | TNFRSF1A | 48470 |
| 7 | CYCS | 247762 | 32 | RAF1 | 80332 | 57 | THY1 | 55638 | 82 | IMMT | 47734 |
| 8 | APP | 233388 | 33 | ENO2 | 79618 | 58 | MEF2C | 54096 | 83 | PSMD12 | 47728 |
| 9 | SNAP25 | 230526 | 34 | ARRB1 | 76960 | 59 | FAU | 53294 | 84 | KPNA2 | 47598 |
| 10 | SNCA | 216470 | 35 | POLR2C | 76364 | 60 | MT2A | 52636 | 85 | CDK7 | 47528 |
| 11 | CAV1 | 203404 | 36 | NME1 | 74914 | 61 | TUBA4A | 52578 | 86 | MDH1 | 47412 |
| 12 | BDNF | 194384 | 37 | XRCC6 | 74458 | 62 | TOP1 | 52474 | 87 | MYO1C | 47172 |
| 13 | CCT7 | 149888 | 38 | MFN2 | 73850 | 63 | HIST1H2AC | 52040 | 88 | TAGLN3 | 46882 |
| 14 | EIF2S1 | 135056 | 39 | HDAC2 | 73482 | 64 | RBM25 | 51778 | 88 | ADK | 46882 |
| 15 | IGF1 | 129624 | 40 | RBFOX1 | 71336 | 65 | PPA1 | 51604 | 90 | NDUFA4 | 46340 |
| 16 | PKM | 115146 | 41 | CARM1 | 71266 | 66 | RPL19 | 51578 | 91 | PRKCZ | 46196 |
| 17 | SOX9 | 114524 | 42 | STAT5A | 69384 | 67 | RPL6 | 50458 | 92 | PPIG | 45822 |
| 18 | PSMD4 | 114234 | 43 | WDR12 | 67926 | 68 | IQGAP1 | 50406 | 93 | WDR37 | 45340 |
| 19 | NHP2 | 106462 | 44 | ATP5C1 | 66476 | 69 | SF3B1 | 50250 | 94 | MRPL15 | 43994 |
| 20 | NTRK2 | 96312 | 45 | ATP6V1A | 66394 | 70 | RRP1 | 49970 | 95 | GNG3 | 43980 |
| 21 | CALM1 | 94602 | 46 | COPS5 | 65308 | 71 | RPS19 | 49956 | 96 | DDX24 | 43456 |
| 22 | ACO2 | 92834 | 47 | CXCR4 | 62898 | 72 | PPP1CB | 49916 | 97 | EPB41 | 43120 |
| 23 | MAP2K1 | 89634 | 48 | KARS | 62538 | 73 | CSNK1D | 49880 | 98 | UBE2V2 | 42936 |
| 24 | ATP5B | 88144 | 49 | RPS7 | 62084 | 74 | NDUFA9 | 49652 | 99 | UTP18 | 42648 |
| 25 | ITPR1 | 88040 | 50 | COL1A1 | 61600 | 75 | ATOX1 | 49624 | 100 | TLN1 | 42232 |

**Supplementary Table S3:** List of Genes Selected by the Feature Selection Algorithms.

| 1. **Lasso** | | | | |
| --- | --- | --- | --- | --- |
|  | **ID** | **fdr_pval** | **pval** | **zval** |
| **1** | SST | 0.000428 | 1.61E-06 | -4.797309 |
| **2** | MTSS1 | 0.003193 | 4.32E-05 | -4.089669 |
| **3** | ATP6V1A | 0.013551 | 4.65E-04 | -3.500023 |
| **4** | RAB27B | 0.013603 | 4.74E-04 | -3.494987 |
| **5** | RRAGB | 0.014223 | 5.17E-04 | -3.471755 |
| **6** | TMBIM4 | 0.016912 | 6.75E-04 | -3.399451 |
| **7** | SLIT3 | 0.01739 | 7.15E-04 | -3.383598 |
| **8** | MFSD9 | 0.017796 | 7.41E-04 | -3.373977 |
| **9** | FCER1G | 0.019547 | 8.80E-04 | -3.326454 |
| **10** | HSPB3 | 0.022379 | 1.10E-03 | -3.262667 |
| **11** | IL27RA | 0.022802 | 1.14E-03 | -3.253059 |
| **12** | PUM1 | 0.028711 | 1.68E-03 | -3.142083 |
| **13** | KCNK7 | 0.037675 | 2.67E-03 | -3.003538 |
| **14** | PDE6H | 0.040896 | 3.06E-03 | -2.961842 |
| **15** | HRG | 0.044847 | 3.63E-03 | -2.908681 |
| **16** | ZC3H15 | 0.038467 | 2.77E-03 | 2.992096 |
| **17** | DSTYK | 0.035 | 2.37E-03 | 3.039378 |
| **18** | CYP4A11 | 0.033186 | 2.14E-03 | 3.070302 |
| **19** | SEMA6A | 0.013551 | 4.65E-04 | 3.500329 |
| **20** | PMEPA1 | 0.00492 | 9.33E-05 | 3.907269 |
| **21** | CBR4 | 0.004041 | 6.72E-05 | 3.986024 |
| **22** | TMEM106C | 0.003451 | 5.15E-05 | 4.048541 |
| **23** | SLC22A18AS | 0.000037 | 5.22E-08 | 5.4438 |
| **24** | CCNC | 0.000021 | 2.48E-08 | 5.574466 |
| **25** | SLC25A46 | 0.000011 | 1.03E-08 | 5.725082 |
| **26** | MT1M | 0.000011 | 9.55E-09 | 5.738541 |
| **27** | KCNE4 | 0.00001 | 7.09E-09 | 5.788827 |

| 1. **Ridge** | | | | |
| --- | --- | --- | --- | --- |
|  | **ID** | **fdr_pval** | **pval** | **zval** |
| **1** | SST | 0.000428 | 1.61E-06 | -4.797309 |
| **2** | PNOC | 0.002478 | 2.84E-05 | -4.185896 |
| **3** | DUSP6 | 0.010701 | 3.23E-04 | -3.596366 |
| **4** | UNC13A | 0.012718 | 4.24E-04 | -3.5246 |
| **5** | NT5M | 0.016421 | 6.48E-04 | -3.410734 |
| **6** | FCER1G | 0.019547 | 8.80E-04 | -3.326454 |
| **7** | FABP3 | 0.01969 | 8.88E-04 | -3.323852 |
| **8** | DNASE1L2 | 0.027851 | 1.57E-03 | -3.160973 |
| **9** | E2F5 | 0.033237 | 2.15E-03 | -3.069378 |
| **10** | ADRA1B | 0.040248 | 2.96E-03 | -2.972287 |
| **11** | PDE6H | 0.040896 | 3.06E-03 | -2.961842 |
| **12** | ACO2 | 0.049981 | 4.34E-03 | -2.852674 |
| **13** | SLC12A9 | 0.046207 | 3.84E-03 | 2.891246 |
| **14** | MKRN3 | 0.046131 | 3.82E-03 | 2.892733 |
| **15** | ZNF639 | 0.042722 | 3.38E-03 | 2.930581 |
| **16** | NEU3 | 0.042324 | 3.32E-03 | 2.936453 |
| **17** | PAX5 | 0.040658 | 3.02E-03 | 2.965505 |
| **18** | MED14 | 0.040248 | 2.96E-03 | 2.972205 |
| **19** | PPIG | 0.040219 | 2.94E-03 | 2.973765 |
| **20** | COLEC12 | 0.039916 | 2.91E-03 | 2.976715 |
| **21** | MYRF | 0.038433 | 2.74E-03 | 2.995116 |
| **22** | FUT6 | 0.036547 | 2.55E-03 | 3.017006 |
| **23** | LTK | 0.03563 | 2.44E-03 | 3.030939 |
| **24** | RHOQ | 0.03506 | 2.38E-03 | 3.03762 |
| **25** | FBXO38 | 0.031749 | 2.00E-03 | 3.090047 |
| **26** | VCAM1 | 0.027624 | 1.55E-03 | 3.165464 |
| **27** | ZNF274 | 0.020959 | 9.84E-04 | 3.294935 |
| **28** | KIFC3 | 0.020185 | 9.23E-04 | 3.312872 |
| **29** | NRIP2 | 0.018771 | 8.23E-04 | 3.344911 |
| **30** | DLGAP2 | 0.018771 | 8.21E-04 | 3.345592 |
| **31** | FDFT1 | 0.017217 | 6.99E-04 | 3.390087 |
| **32** | COPE | 0.015371 | 5.86E-04 | 3.437906 |
| **33** | CDC25A | 0.014655 | 5.47E-04 | 3.45684 |
| **34** | SEMA6A | 0.013551 | 4.65E-04 | 3.500329 |
| **35** | PNMA3 | 0.011237 | 3.44E-04 | 3.579582 |
| **36** | TMF1 | 0.009718 | 2.76E-04 | 3.636405 |
| **37** | PTAFR | 0.008854 | 2.36E-04 | 3.67675 |
| **38** | LTF | 0.00742 | 1.78E-04 | 3.747726 |
| **39** | MAP1B | 0.003636 | 5.85E-05 | 4.018929 |
| **40** | FOXD1 | 0.002311 | 2.56E-05 | 4.209259 |
| **41** | SLC25A46 | 0.000011 | 1.03E-08 | 5.725082 |
